# Bayesian refinement of protein structures and ensembles against SAXS data using molecular dynamics

**DOI:** 10.1371/journal.pcbi.1005800

**Published:** 2017-10-18

**Authors:** Roman Shevchuk, Jochen S. Hub

**Affiliations:** 1 Institute for Microbiology and Genetics, University of Göttingen, Göttingen, Germany; 2 Göttingen Center for Molecular Biosciences (GZMB), University of Goettingen, Goettingen, Germany; US Army Medical Research and Materiel Command, UNITED STATES

## Abstract

Small-angle X-ray scattering is an increasingly popular technique used to detect protein structures and ensembles in solution. However, the refinement of structures and ensembles against SAXS data is often ambiguous due to the low information content of SAXS data, unknown systematic errors, and unknown scattering contributions from the solvent. We offer a solution to such problems by combining Bayesian inference with all-atom molecular dynamics simulations and explicit-solvent SAXS calculations. The Bayesian formulation correctly weights the SAXS data versus prior physical knowledge, it quantifies the precision or ambiguity of fitted structures and ensembles, and it accounts for unknown systematic errors due to poor buffer matching. The method further provides a probabilistic criterion for identifying the number of states required to explain the SAXS data. The method is validated by refining ensembles of a periplasmic binding protein against calculated SAXS curves. Subsequently, we derive the solution ensembles of the eukaryotic chaperone heat shock protein 90 (Hsp90) against experimental SAXS data. We find that the SAXS data of the apo state of Hsp90 is compatible with a single wide-open conformation, whereas the SAXS data of Hsp90 bound to ATP or to an ATP-analogue strongly suggest heterogenous ensembles of a closed and a wide-open state.

## Author summary

In solution, many proteins adopt ensembles of multiple distinct states. The relative concentrations of the states are tightly controlled by factors such as pH, phosphorylation, or ligand binding, and a misbalance between the states underlies diseases such as cancer or neurodegeneration. However, detecting protein ensembles in experimental data has remained challenging. We present a statistically founded procedure for refining protein structures and ensembles against X-ray solution scattering data by combining atomistic simulations with Bayesian inference.

## Introduction

Proteins are dynamic nanomachines that often populate heterogeneous ensembles of multiple distinct structural states. Controlling the relative population of such states is pivotal for the correct functioning of biological cells, and any misbalance between states may lead to severe conditions such as cancer or neurodegeneration. Detecting, understanding, and manipulating heterogeneous protein ensembles has therefore remained a central goal of molecular biophysics [1].

Deriving solution ensembles of proteins from structural experimental data has remained challenging, mainly because the information content of the data is typically insufficient to define all degrees of freedom of the ensemble [2, 3]. Consequently, upon fitting of structures or ensembles against experimental data, the data must be complemented by a physical model that restrains the protein into physically reasonable conformations, thereby reducing the risk of overfitting the model. Bayesian inference provides a route founded on probability theory for combining experimental data with physical models [4]. Applied to structure determination, Bayesian inference may become computationally expensive and technically challenging since it requires explicit sampling of the conformational space of the protein. However, it also holds a number of key advances over more simple optimization algorithms, as it provides statistically founded procedures (i) to weight the experimental data versus prior physical knowledge, and (ii) to quantify the uncertainty (or ambiguity) of the fitted structural model [5]. Due to its probabilistic rigor, Bayesian inference has been gaining increased popularity in various fields of biophysics, and it has hence been successfully applied for the refinement of structures against restraints from NMR, EPR, cryo-EM, and single-particle X-ray diffraction [5–11]. Following the pioneering work by Rieping et al., we refer to structural modeling based on Bayesian statistics as ‘inferential structure determination’ (ISD) [6].

**Fig 1:**
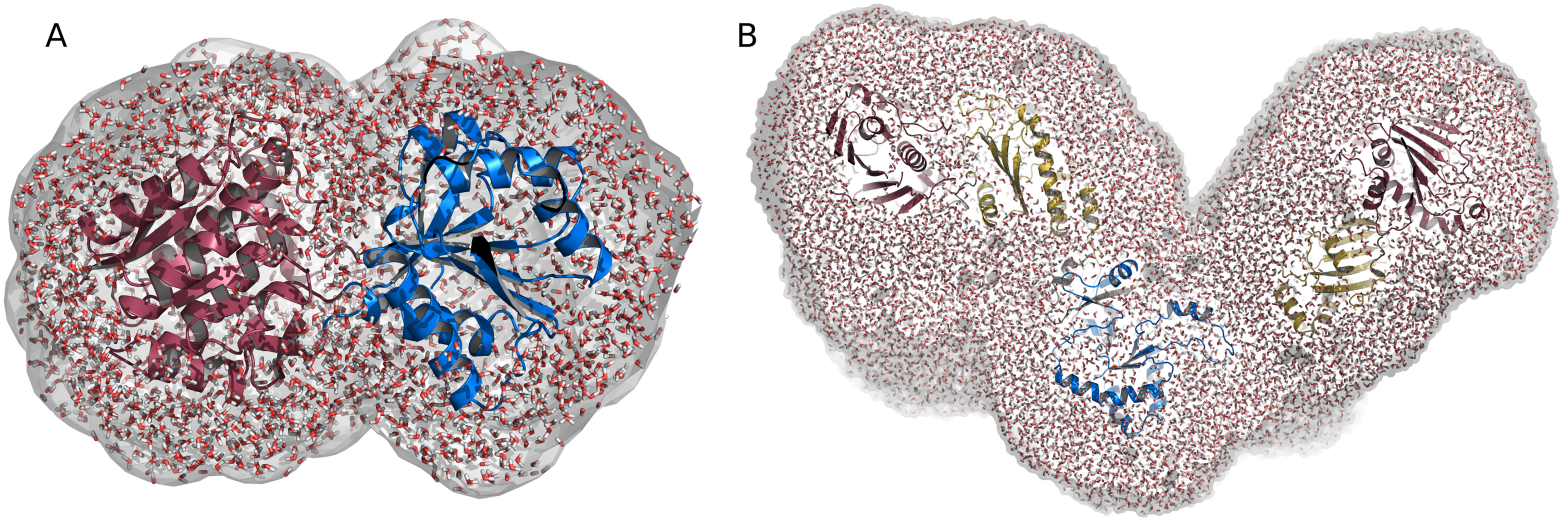
Envelopes of (A) leucine binding protein (LBP) and (B) heat shock protein 90 (Hsp90), illustrated as a white surface. Water molecules (red/white sticks) inside the envelope contributed to the explicit-solvent calculations used to compute the SAXS curves and the SAXS curve gradients, as required for the refinement simulations. (A) The N- and C-terminal domains of LBP are shown in red and blue cartoon representation, respectively. (B) The N-terminal, middle, and C-terminal domains of Hsp90 are shown in red, yellow, and blue cartoon representation, respectively.

Small-angle X-ray scattering (SAXS) is an increasing popular method that is in principle capable of detecting biomolecular structures and ensembles in solution [12, 13]. However, due to the low information content of SAXS data, refining structures or ensembles without overfitting poses a major challenge. For the refinement of individual structures against SAXS data, two routes have been suggested to reduce the risk of overfitting: first, during refinement, nearly all degrees of freedom of the biomolecule are constrained, leading to methods such as rigid-body modelling or normal mode fitting [14–17]. Second, physical information may be added to the low-information SAXS data, for instance by coupling a force field-based molecular dynamics (MD) simulation to the data with an energetic restraint [18–20]. Here, we follow the second route, building upon our method of SAXS-driven MD simulations [18]. SAXS-driven MD simulations drive biomolecular structures into conformations that are compatible with the data, using a differentiable harmonic restraint to the data. Critically, the method employs explicit-solvent calculations for predicting SAXS curves from the simulations frames, which were shown to provide accurate prediction for small and wide angles without the need of adjusting fitting parameters for the hydration layer or excluded solvent (see Fig 1) [21, 22]. In other words, the method uses a highly accurate and predictive ‘forward model’. However, as formulated previously, the method was not Bayesian and, consequently, did not yet benefit from advantages of ISD-related approaches (see above).

Many methods for the refinement of heterogenous ensembles against experimental data follow a “sample-and-select” strategy [23]. Accordingly, first, an ensemble is proposed by sampling from a computationally efficient physical model, such as a coarse-grained force field. Second, a limited number of structures or clusters are picked from the proposed ensemble. Third, the weights of the structures or clusters are modified in a statistically meaningful manner until the data back-calculated from the refined ensemble agrees with the given experimental data. Examples of this strategy in the context SAXS are the EROS, BSS-SAXS, and EOM methods [24–26], yet a number of related approaches have been suggested ([23] and references therein). Interesting recent developments proposed the Bayesian derivation of continuous ensembles from experimental data, including a development for SAXS data [27, 28].

In this work, we take an alternative approach for the refinement of ensembles against SAXS data. First, following the ISD approach, we embed SAXS-driven MD simulations into a Bayesian inference framework. Hence, we derive *posterior* distributions of protein structures in the light of the SAXS data and the applied force field. Because we simulate with a physically accurate all-atom force field, we employ an accurate and informative *prior* of protein structures. Additional unknown parameters, termed nuisance parameters in context of ISD [6], are not chosen ad hoc but instead estimated simultaneously with the protein structures. Specifically, two remaining fitting parameters as well as a systematic uncertainty due to the buffer subtraction are taken as nuisance parameters. Second, we extend the concept of ISD towards an ensemble of a small number of structural states, allowing us to estimate the structural weights simultaneously with the structures and the nuisance parameters. In combination, due to the commitment to Bayesian inference, the new method provides statistically founded confidence intervals for both the structures *and* the structural weights. In addition, we show that the posterior distribution for the structural weights can be used as a criterion for detecting the number of states in the ensemble that is required to explain the data.

### Bayesian interpretation of SAXS data: Goal of the method

We consider proteins that adopt an ensemble of a small number of distinct states. Typical examples would be proteins that exist in a mixture of active and inactive states, apo and holo states, or in a mixture of a few states along a more complex conformational cycle. We aim to derive the coordinates **R** = (**R**_1_, …, **R**_*N*_) as well as the relative weights (or concentrations) **w** = (*w*_1_, …, *w*_*N*_) of the states from given experimental SAXS data, where *N* is the number of states. Hence, the term ‘ensemble’ does not refer to the thermodynamic ensemble, but instead to a specific set (**R**, **w**). Notably, since the ensemble reduces to a single structure by setting *N* = 1, Bayesian structure refinement (instead of ensemble refinement) is contained in the method presented here as a special case.

### Posterior distribution of the ensemble

Since the number of independent data points in a SAXS curve is much smaller than the number of degrees of freedom of any protein, it is unlikely that only a single ensemble (**R**, **w**) fits the SAXS data, but instead a wide range of ensembles are typically compatible with the data. A statistically founded procedure to infer the ensemble from the data can be formulated as the conditional probability *p*(**R**, **w**, *θ*|*D*, *K*) that quantifies the plausibility of the ensemble (**R**, **w**) in the light of the SAXS data *D* and prior physical knowledge *K* [4]. The symbol *θ* summarizes nuisance parameters, which are of limited interest but required for evaluating whether the ensemble (**R**, **w**) is compatible with the data *D* (see below). The posterior distribution is most conveniently evaluated using Bayes’ theorem:

$$\begin{array}{c} p(R,w,\theta |D,K)\propto L(D|R,w,\theta ,K)\pi (R|K)\pi (w|K)\pi (\theta |K). \end{array}$$ (1)

Here, *L*(*D*|**R**, **w**, *θ*, *K*) denotes the likelihood that the data *D* were measured given the ensemble (**R**, **w**) and nuisance parameters *θ*. The functions *π*(**R**|*K*), *π*(**w**|*K*), and *π*(*θ*|*K*) denote the prior distributions of possible protein conformations, weights, and nuisance parameters. Due to the low information content of SAXS data, *L*(*D*|**R**, **w**, *θ*, *K*) provides only limited information, i.e., *L* is a wide function of *R*. Hence, in order to draw structural conclusions from the data, that is, to arrive at a reasonably narrow posterior distribution, it is critical to impose an informative tight prior *π*(**R**|*K*) of protein conformations, which is here achieved by applying an accurate physical model. In the method presented here, the prior *π*(**R**|*K*) is naturally given through an unbiased MD simulation, where *K* represents the physical laws and the force field underlying the MD simulation.

### Accounting for unknown systematic errors

Formulating a likelihood function *L* for SAXS refinement is not straightforward because experimental SAXS data report purely statistical errors, whereas systematic errors, for instance due to poor buffer matching, are typically unknown. For data recorded at modern single-photon counting detectors, systematic error may dominate the overall uncertainty, suggesting that systematic errors strongly contribute to the likelihood *L*. In addition, for comparing experimental with calculated SAXS curves, free fitting parameters must be adjusted [21, 29–31]. Since both the systematic errors and fitting parameters are a priori unknown, a full Bayesian treatment requires that those parameter are simultaneously estimated with the structures and weights. Hence, systematic errors as well as fitting parameters are treated as nuisance parameters *θ* in the present method.

In practice, one is mainly interested in the ensemble (**R**, **w**), but not in the nuisance parameters *θ*. The statistically correct way of reducing the general posterior *p* in Eq 1 to the posterior of the structural ensemble is to marginalize out the nuisance parameters,

$$\begin{array}{c} p(R,w|D,K)=\int d\theta \;p(R,w,\theta |D,K). \end{array}$$ (2)

In our method, the fitting parameters are marginalized out analytically at the level of the likelihood, whereas systematic errors are explicitly sampled and marginalized out numerically (Methods and materials). To visualize the high-dimensional *p*(**R**, **w**|*D*, *K*), the posterior may be further projected onto intuitively important coordinates, such as the distance between two protein domains, the radius of gyration of the protein, or the weight of an interesting state.

### Energy analogs

By taking the negative logarithm of the posterior, Eq 1 takes the form of a hybrid energy that is commonly applied for structure refinement [5, 9], yet corrected with the contributions from the prior distributions:

$$\begin{array}{c} E_{\text{hybrid}}=V_{\text{ff}}\left(R,K\right)+E_{\text{exp}}\left(R,w,\theta ,D,K\right)-\beta^{-1}\ln\left[\pi \left(w|K\right)\;\pi \left(\theta |K\right)\right]. \end{array}$$ (3)

Here, the posterior was identified with a hybrid energy *E*_hybrid_ = −*β*^−1^ ln *p*(**R**, **w**, *θ*|*D*, *K*), where *β* denotes the inverse temperature. The prior for the protein structures is taken from the MD force field energy as *V*_ff_(**R**, *K*) = −*β*^−1^ ln *π*(**R**|*K*), after marginalizing out the solvent coordinates (see Methods and materials). The experiment-derived energy is given via the likelihood, *E*_exp_ = −*β*^−1^ ln *L*, adding an energetic penalty if the SAXS curve calculated from the ensemble (**R**, **w**) is incompatible with the data *D*.

### Sampling the posterior distribution

Having translated the probabilities into energies, all parameters can be sampled using established methods. Accordingly, sampling of protein structures **R** is conducted using Newtonian dynamics. Here, the force on atom *ℓ* is given via gradients of the hybrid energy with respect to the atomic positions, **F***_ℓ_* = −∇*_ℓ_* *E*_hybrid_, evaluated at fixed **w** and fixed nuisance parameters. The fitting parameters, as shown below, are marginalized analytically at the level of the likelihood. The remaining nuisance parameter, namely the systematic error *σ*_buf_, as well as the weights **w** are sampled using Gibbs sampling, that is, Monte-Carlo moves at fixed protein coordinates **R**. Calculations of the SAXS intensity and intensity gradients from (**R**, **w**), as required for sampling the posterior, were conducted with the explicit-solvent algorithms established previously [18, 21], taking accurate atomistic models for both the hydration layer and the excluded solvent (Fig 1). Details on the likelihood function, assumed priors, force calculations, and sampling algorithms are provided in the Methods and Materials.

### A probabilistic criterion for choosing the number of states

The weights **w** are normalized and have non-negative elements, i.e., the relevant weight space is given by the (*N* − 1)-simplex. Sampling of the weight space was accelerated using umbrella sampling along the weights [32]. This is computationally convenient because it allows calculation of the posterior from a set of short independent simulations. More critically, this allows us to compute the posterior for the complete weight space, including the “edge” of the simplex, where at least one of the weights *w*_*j*_ is zero (1 ≤ *j* ≤ *N*). However, note that weight vectors **w** with elements equal to zero specify smaller ensembles with a reduced number of states. Consequently, the posterior of an ensemble of *N* states includes all smaller ensembles as a special case, thereby proving a probabilistic criterion for choosing the number of states required to explain the experimental data: if the posterior peaks at the edge of the simplex, a smaller ensemble provides a plausible model; in turn, if the posterior near the edge is small compared to the posterior’s maximum, a smaller ensemble is implausible.

## Results

In the following, Bayesian ensemble refinement is demonstrated for two test proteins: leucine binding protein (LBP) using calculated SAXS data and heat shock protein 90 (Hsp90) using experimental SAXS data. We assumed that both proteins adopt a two-state ensemble of an open and a closed structure (*N* = 2). We further assume that the closed structure is known, whereas (i) the coordinates of the open structure as well as (ii) the relative open/closed weights are simultaneously refined against SAXS data. Such scenarios are quite common, as a compact holo or ground state structure might be accessible to X-ray crystallography, whereas more flexible apo or excited state structures often do not crystallize. Applying the method proposed here to larger ensembles of *N* > 2 is conceptually possible but beyond the scope of this article. With increasing number of states *N*, due to increasing number of required umbrella windows, the computational cost would scale exponentially with *N* − 1.

### Leucine binding protein (LBP)

**Fig 2:**
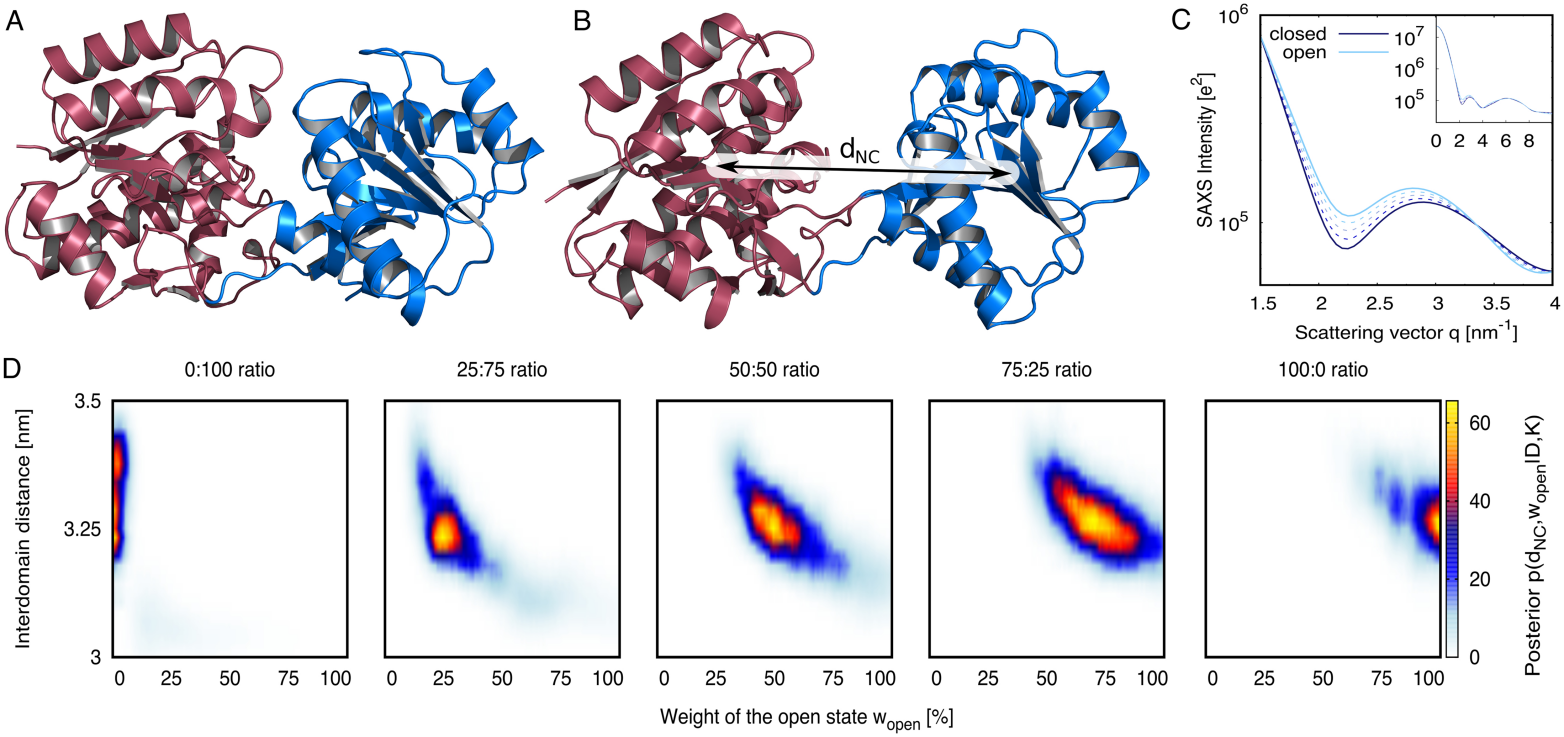
Bayesian ensemble refinement of leucine binding protein (LBP). (A) Cartoon representation of LBP in closed and (B) open states. N- and C-terminal domains are colored in red and blue, respectively. (C) Solid lines: Computed SAXS curves of the open (light blue) and closed state (dark blue). Dashed lines: SAXS curves of open/closed heterogeneous ensembles, computed with open/closed weights of 25:75, 50:50, and 75:25. Inset: Complete SAXS curves up to *q* = 10 nm^−1^. Large figure: closeup view highlighting the differences between the SAXS curves. (D) Posterior distribution of the refined two-state ensemble, projected onto the weight *w*_open_ and the interdomain distance *d*_NC_ of the open state. The one-dimensional marginalized distributions are shown in Fig 3A and S1 Fig.

LBP is a typical representative of the superfamily of periplasmic binding proteins involved in chemotaxis and solute uptake over membranes [33]. LBP is a well-characterized two-domain protein, exhibiting a transition from an open (apo) to a closed (holo) state triggered by ligand binding (Fig 2A/2B) [34, 35]. Free simulations of the closed and open state suggested center-of-mass distances between the N- and C-terminal domains of ∼3 nm and 3.25 nm, respectively, which is compatible with experimental SAXS data of the homologous LIVBP [18]. We theoretically computed SAXS curves of the open and closed states (Fig 2C, solid lines), as well as linear combinations, thereby modeling SAXS data from heterogeneous ensembles of known open/closed weights of 0:100, 25:75, 50:50, 75:25, and 100:0 (Fig 2C, dashed lines).

The posteriors of the ensembles *p*(**R**, **w**|*D*, *K*) refined against these five SAXS curves are presented in Fig 2D. To visualize the high-dimensional posterior, we projected the posterior onto two characteristic coordinates: (i) the weight of the open state *w*_open_, implying the weight (1 − *w*_open_) for the closed state, and (ii) the interdomain distance *d*_NC_ of the open state (illustrated in Fig 2B). Evidently, all derived posterior distributions peak at the correct *w*_open_. In addition, the posteriors refined against SAXS curves of non-zero open-state content (Fig 2D, four right panels) peak at the physically correct interdomain distance of ∼3.25 nm (Fig 2, see also the marginalized posteriors in S1A Fig). In addition, the RMSD to the mean open structure taken from umbrella simulations, restrained to weights at the maxima of the respective posterior, reveals that the refinement simulations rapidly approach the correct open state (S5 Fig). These findings demonstrate that the MD simulations were capable of translating the information in the SAXS curve into the underlying heterogeneous open/closed ensemble.

**Fig 3:**
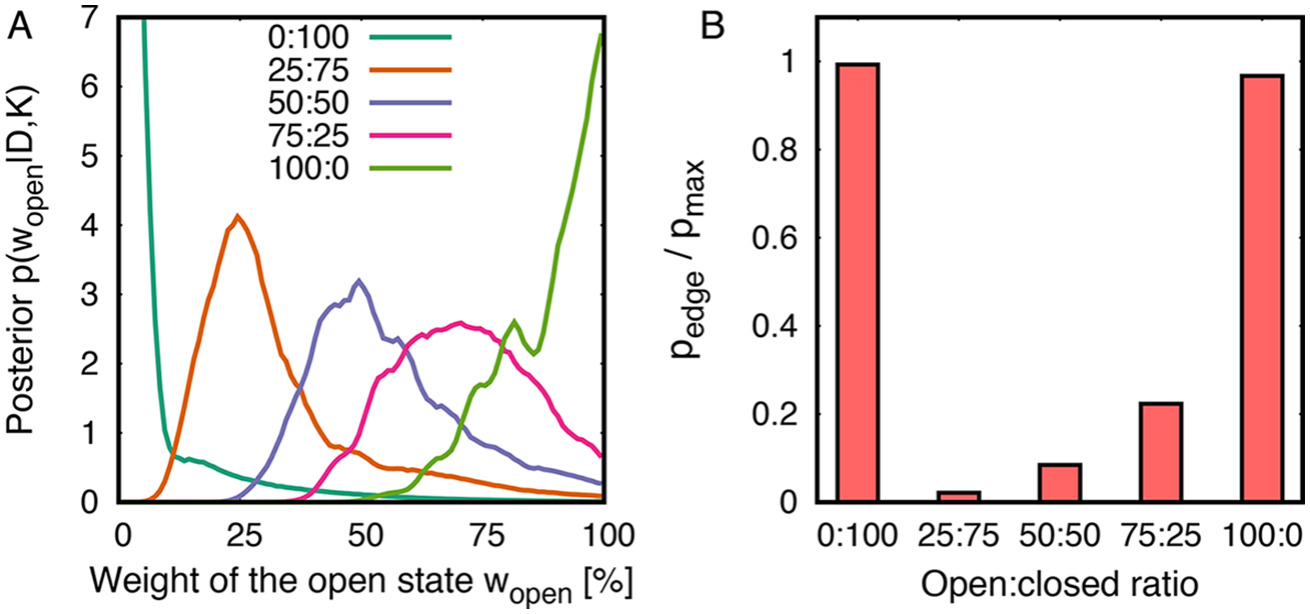
(A) Marginalized posterior of the weight of the open state from refinement of leucine binding protein (LBP). (B) Odds that a single state vs. a two-state ensemble underlies the SAXS curves, presented as *p*_edge_/*p*_max_. For details, see text.

The width of the posteriors rigorously quantify the degree of structural knowledge that can (and cannot) be inferred from the SAXS curve, i.e., the posteriors quantify the ambiguity of the refined ensemble. For the LBP ensemble refinement, the marginalized posteriors suggest 65% confidence intervals (CI) for *w*_open_ and *d*_NC_ in the order of ±15% and ±0.07 nm, respectively (Fig 3A and S1A Fig, S1 and S2 Tables). In addition, the posteriors in Fig 2D suggest some correlation between *w*_open_ and *d*_NC_, as apparent from the posterior’s diagonal elongated shapes, suggesting that the SAXS curves are compatible with an increased *w*_open_ given that the open state is modeled more compact.

#### Single-state or two-state ensemble?

Fig 3A presents the posterior distributions *p*(*w*_open_|*D*, *K*), as derived from the two-dimensional posteriors *p*(*w*_open_, *d*_NC_|*D*, *K*) (Fig 2D) by marginalizing out the interdomain distance *d*_NC_. As described in the Theory section, the odds that a single state versus a two-state ensemble underlie the SAXS data is quantified by *p*(*w*_open_|*D*, *K*) at the “edge” of the weight space, at *w*_open_ = 0 and *w*_open_ = 1, as compared to *p*(*w*_open_|*D*, *K*) at intermediate *w*_open_. Fig 3B compares the posterior maximum at the edge *p*_edge_ with the posterior maximum in the entire weights space *p*_max_, plotted as *p*_edge_/*p*_max_. Evidently, the posteriors refined against SAXS curves representing purely the closed or purely the open state exhibit a peak at the edge, thus recovering that a single state is sufficient to explain this data (Fig 3A/3B, 0:100 and 100:0). In contrast, posteriors refined against SAXS curves of heterogeneous ensembles are small at the edge and instead peak at intermediate *w*_open_ (Fig 3A/3B, 25:75, 50:50, 75:25). Hence, our method recovers that a single state is highly implausible in the light of these SAXS curves and the force field, and that instead two states are required to explain the data.

It is instructive to compare the two-state refinement presented in Figs 2 and 3 with an attempt to interpret the five SAXS curves of Fig 2D by a single state. To this end, we refined a single state (*N* = 1) against each of the five computed SAXS curves. As expected, refining a single state against the SAXS curves of purely the closed or purely the open state (100:0 or 0:100) recovers the correct interdomain distances of ∼3.0 nm and ∼3.25 nm for the closed and open states, respectively (S1B Fig, S3 Table). In contrast, refining a single state against SAXS curves that, in truth, represent a heterogeneous open/closed ensemble (25:75, 50:50, 75:25) leads to a misinterpretation of the SAXS data in terms of intermediate partially open states (S1B Fig, S3 Table), contrasting the fact that such intermediate states are hardly populated in a free microsecond-long simulation. Critically, the fitted SAXS curves well match the target curves, suggesting that a visual inspection of the fitted curves is insufficient to reveal that such partially open states are a misinterpretation (S2 Fig). Hence, an analysis similar to the Bayesian inference on the number of states, as presented in Fig 3, is indeed required to detect the correct number of states from the SAXS curve.

### Heat shock protein 90

**Fig 4:**
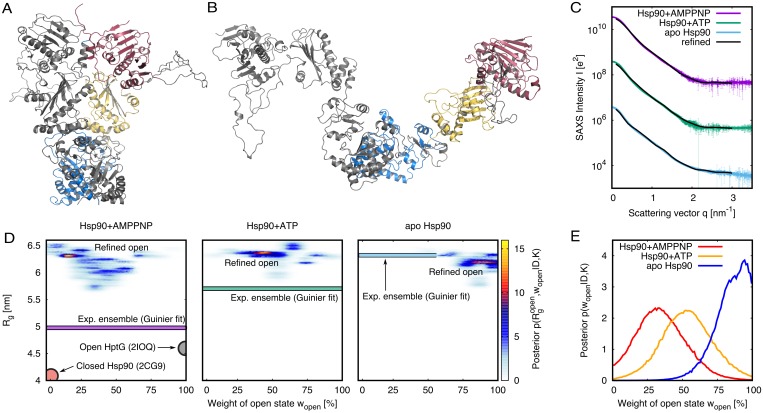
Bayesian ensemble refinement of Hsp90. (A/B) Cartoon representation of Hsp90, the first protomer shown in grey, and the second protomer in color. C-terminal, middle, and N-terminal domains are shown in blue, yellow, and red, respectively. (A) Closed state, modelled from the 2CG9 structure [36]. (B) Open state, refined against SAXS data. (C) Experimental SAXS curves (colored lines, taken from ref. [37]) and calculated SAXS curves (black) computed from the refined ensembles. For clarity, curves for AMPPNP- and ATP-bound states were vertically offset, and experimental data points with very large errors were removed. (D) Posterior distribution of refined Hsp90 ensemble plotted as function of the weight (*w*_open_) and radius of gyration ($R_{g}^{\text{open}}$) of the open state. For reference, The radius of gyration *R*_*g*_ of the closed Hsp90 structure (2CG9) and of the partially open *E. coli* HptG structure (2IOQ) are indicated as pale red and grey circles [36, 38]. In addition, *R*_*g*_ of the experimental ensembles, taken from Guinier fits to the SAXS curves in (C), are indicated as colored bars (color coding according to C). (E) Posterior of *w*_open_, computed from the maps in (D) by marginalizing out *R*_*g*_.

Hsp90 is a chaperone that interacts with more than 200 proteins in eucaryotic cells [39–42]. It constitutes up to 2% of the cellular protein mass [43]. Since many client proteins of Hsp90 are oncogenic, Hsp90 has been suggested as a promising target for anti-cancer therapies [44, 45]. Structurally, Hsp90 is a homodimer, where each protomer contains three domains: an N-terminal domain with the ATP binding site, a middle domain forming the interaction sites for client proteins, and a C-terminal domain responsible for Hsp90 dimerization (Fig 4A). Crystallographic, cryo-EM, and SAXS studies established that Hsp90 carries out large-scale conformational transitions between a V-shaped open state and a compact closed state, controlled by binding of ATP, ATP analogues, and client proteins [38, 46]. However, ligands do not dictate a single well-defined state, but instead merely shift the equilibria of heterogeneous ensembles between open and closed conformations [47, 48]. Only recently it was found that sufficient time spent in the open state is crucial for correct Hsp90 functioning, highlighting the importance of controlling the open/closed equilibria of the chaperone [49].

Based on experimental SAXS data of yeast Hsp90 in the apo state, Hsp90 bound to the slowly hydrolyzing ATP-analogue AMPPNP, and Hsp90 bound to ATP (Fig 4C, colored curves [37]), we derived heterogeneous solution ensembles of the Hsp90 dimer using Bayesian ensemble refinement. Hsp90 ensembles were modeled as two-state ensemble of (i) the closed state, taken from the yeast crystal structure (Fig 4A), and (ii) an initially unknown open state. Starting the simulations from a nearly closed conformation, both the structure and the relative weight *w*_open_ of the open state were simultaneously refined against the SAXS data. The SAXS curves of the refined two-state ensembles exhibited reasonable agreement with the experimental curves (Fig 4C).

**Fig 5:**
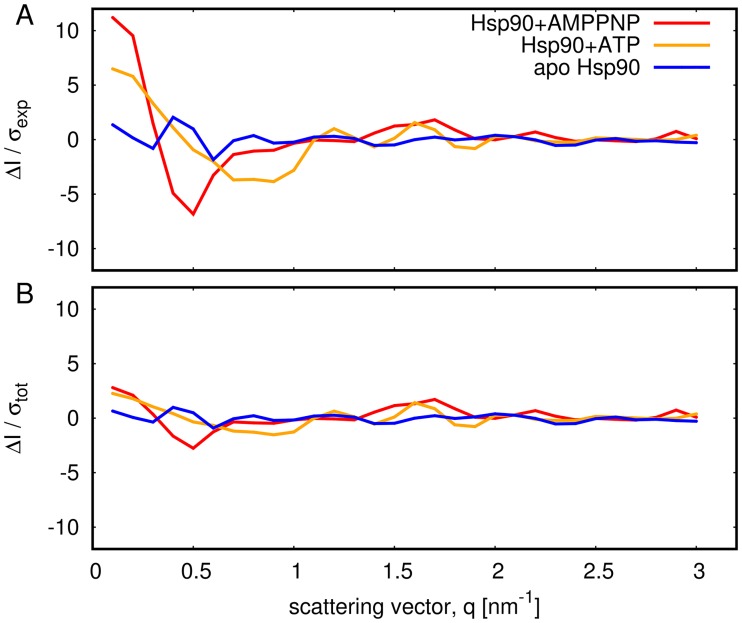
Residuals between the calculated SAXS curves and the experimental SAXS curves, evaluated at the *q*-points applied during the refinement simulations of Hsp90 (color code see legend). (A) Residuals Δ*I*/*σ*_exp_ normalized with respect to purely statistical experimental errors *σ*_exp_. Large residuals at low *q* reflect that the MD force field prohibited structures that would accurately match the data within statistical errors. (B) Residuals Δ*I*(*q*)/*σ*_tot_, where *σ*_tot_ denotes the total error including both statistical and estimated systematic errors (see Methods for details). The reduced residuals compared to panel (A) reflect that the Bayesian analysis suggested substantial systematic errors as the most plausible explanation for discrepancies between calculated and experimental SAXS curves.

The residuals between calculated and experimental SAXS curves are analyzed in Fig 5. Here, panel (A) shows the residuals normalized purely by the statistical experimental errors, Δ*I*(*q*)/*σ*_exp_. The large residuals at low *q* (Fig 5A, red and yellow) reflect that the MD force field did not allow conformations that accurately fullfil the data within statistical experimental errors, possibly because accurately reproducing the data would require an energetically unfavorable conformational transition (such as partial unfolding). In other words, the Bayesian analysis revealed that, in the light of the force field, substantial systematic errors at low *q* are more plausible than an ensemble that accurately matches the experimental data. Indeed, as shown in Fig 5B the residuals normalized with respect to the total errors including statistical *and* systematic errors, Δ*I*(*q*)/*σ*_tot_, reveal reasonably low values over the entire *q*-range. As outlined in the Methods, we modelled systematic errors as a consequence of poor buffer matching, but the analysis can not exclude other sources of remaining discrepancies such as a small fraction of aggregated Hsp90. Further, in this work, we can not fully exclude the possibility that a more continuous ensemble, as supported by recent Förster resonance energy transfer (FRET) study [50], might provide a more accurate description of the experimental conditions.

**Fig 6:**
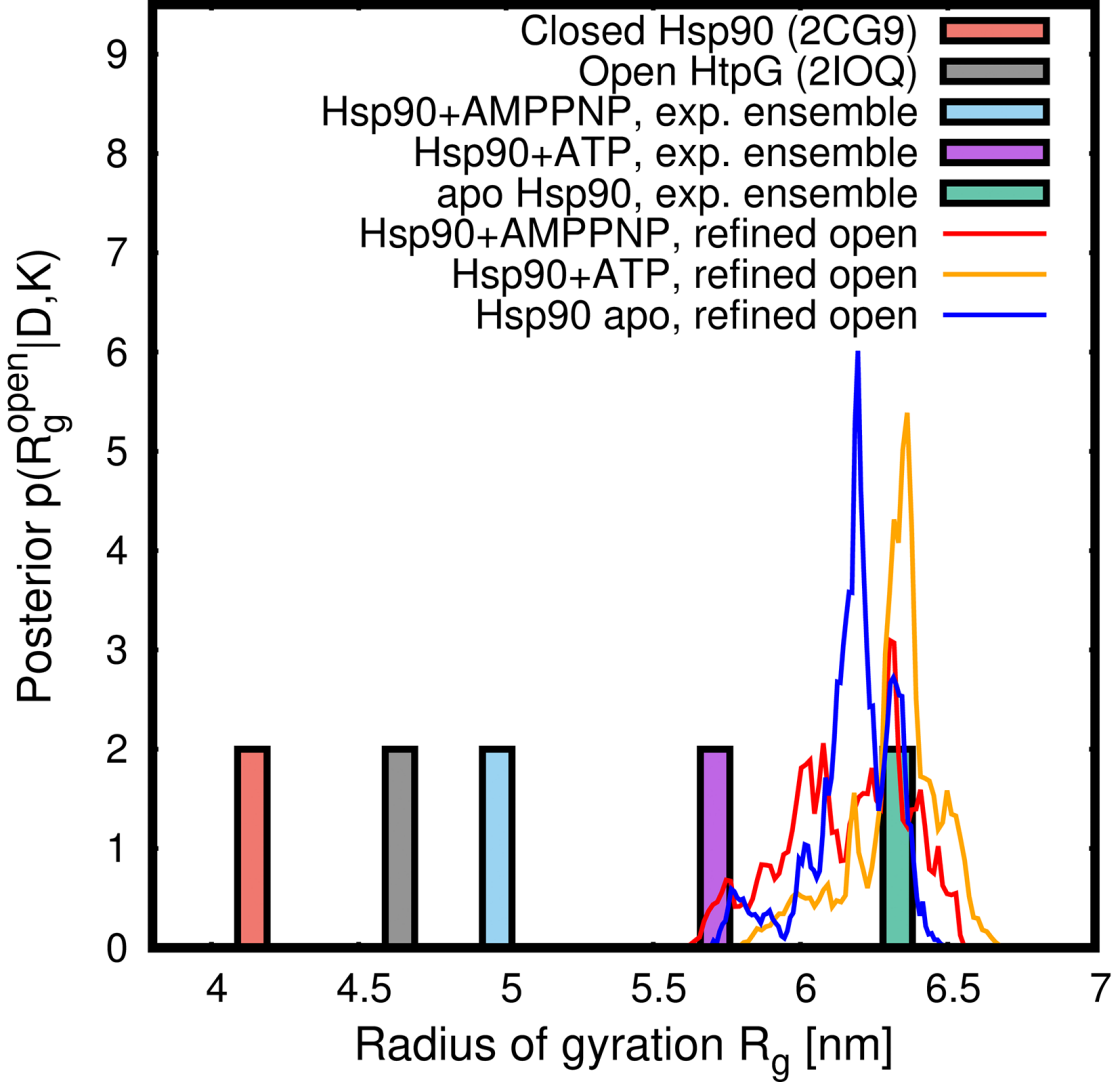
Lines: Marginalized posteriors of the radius of gyration $R_{g}^{\text{open}}$ of the refined open state from two-state refinement of Hsp90+AMPPNP, Hsp90+ATP, and apo Hsp90 (for color code, see legend). The posteriors characterize a wide open conformation. For comparison, pale green, purple, and pale blue bars indicate the *R*_*g*_ values for the ensembles of Hsp90+AMPPNP, Hsp90+ATP, and apo Hsp90, respectively, estimated from a Guinier fit to the experimental data [37]. The pale red bar indicates *R*_*g*_ of the closed crystal structure of Hsp90 (2CG9), and the grey bar indicates *R*_*g*_ of the partially open crystal structure of *E. coli* HptG (2IOQ) [36, 38].

Fig 4D presents the posterior distributions *p*(**R**, **w**|*D*, *K*) of the Hsp90 ensembles, projected onto two intuitive degrees of freedom: (i) the weight *w*_open_ of the open state, implying the weight (1 − *w*_open_) for the closed state, and (ii) the radius of gyration $R_{g}^{\text{open}}$ of the refined open state, which naturally quantifies the degree of opening of the open state. The marginal posteriors $p(R_{g}^{\text{open}}|D,K)$ for the three ensembles, obtained by marginalizing the posteriors in Fig 4D with respect to *w*_open_, are presented in Fig 6 as colored lines. Evidently, the refined structures of the open state were similar in all three ensembles, exhibiting large $R_{g}^{\text{open}}$ values of ∼6.3nm. These $R_{g}^{\text{open}}$ values are ∼1.3 nm and ∼1.7 nm larger than the radii of gyration of the open form of the bacterial HtpG homologue in the crystal and in solution environment, respectively [38, 48], but they are compatible with previously reported open conformations of eukaryotic apo Hsp90s [51]. Hence, the open structures of the three refined open/closed heterogeneous ensembles are characterizing by a wide open conformation, as visualized in Fig 4B.

Fig 4E presents the marginal posteriors of the weight of the open state, *p*(*w*_open_|*D*, *K*), obtained by marginalizing the posteriors in Fig 4D with respect to $R_{g}^{\text{open}}$. Evidently, *w*_open_ strongly differs between the three ensembles. The posteriors suggest closed:open populations of 68:32 and 52:48 for the AMPPNP- and ATP-bound states, respectively, with 65% confidence intervals of ±18% (S4 Table). Hence, for the AMPPNP- and ATP-bound states, a model of a single state is very implausible in the light of the MD force field and the SAXS data. These findings resemble results from rigid-body SAXS modeling of a bacterial HtpG homologue that suggested heterogeneous closed/open ensembles in the AMPPNP-bound state, yet without providing confidence intervals [48]. For the Hsp90 apo state, the posterior suggests that *w*_open_ is with 65% confidence within the interval [78%,100%], suggesting that a single open state as well as a heterogeneous ensemble with a large *w*_open_ are both compatible with the SAXS data and the MD force field.

## Discussion

We have presented a method for the refinement of a single protein structure or of an ensemble of structures against SAXS data, applicable to ensembles of a small number of distinct states. By combining Bayesian inference with atomistic MD simulations, the method is capable of inferring the structures and structural weights that gave rise to the SAXS data. The method does not merely derive a single “best fit” against the experimental data, but instead provides the joint posterior distribution of structures and weights, thus quantifying the plausibility of all possible structures and ensembles in the light of data *D* and physical knowledge *K*. The width of the posteriors yield confidence intervals founded on probability theory for both the structures and the weights, that is, the method quantifies the precision of the refined ensemble. Such reliable confidence intervals are required for deciding whether a structural model is convincingly supported by available SAXS data, or whether additional data are needed to unambiguously prove a model. We stress that the confidence intervals derived here should not be confused with the spread of “best fits” obtained by multiple repetitions of an optimization algorithm, as common, for instance, when fitting low-resolution bead models against SAXS data [52]. Repeated best fits test the convergence of the optimization algorithm but do not provide a statistically founded confidence interval.

Since we enforced exhaustive sampling of the weight space using umbrella sampling, the posterior includes smaller ensembles with a reduced number of states as a special case, as given by weight vectors **w** with one or multiple zero elements. We showed that this feature provides a rigorous criterion for deciding on the number of states required to explain the experimental data. For the apo state of Hsp90, we found that the SAXS data is compatible with a single open state, as well as with a heterogenous open-closed ensemble with a large weight of the open state. In contrast, for the AMPPNP- and ATP-bound states of Hsp90, we found that a single state is unlikely in the light of the SAXS data and the MD force field, whereas a model of two states provides a much more plausible model. Critically, Bayesian inference further allows us to assign a confidence to these qualitative statements. Namely, the odds that a single state underlies the SAXS data is 80% for the apo state, 20% AMPPNP-bound, and 6% for the ATP-bound state. As such, the researcher may decide whether such confidence is sufficient to decide on the number of states, or whether additional data, e.g. from FRET, should be included to further increase the confidence on the number of states [53].

A property of Bayesian methods is that the computed posterior depends on the chosen priors. For Bayesian SAXS refinement, the posterior *p*(**R**, **w**) most critically depends on the prior for protein conformations *π*(**R**, *K*), which is given through the applied force field. In this work, we applied a physically accurate all-atom force field, which provides a more accurate description of the energy landscape as compared to rigid-body or coarse-grained force fields. However, despite major force field improvements in recent years [54], it can not be excluded that certain force fields bias the refinement simulations towards unphysical states, in particular for proteins with large disordered regions [55]. Hence, we recommend to use the most recent and best validated force fields.

Depending on the size of the system and the inherent autocorrelation times, exhaustive sampling of the posterior may become challenging. Due to the use of umbrella sampling along *w*_open_, we here observed rapid convergence of the marginalized posterior *p*(*w*_open_|*D*, *K*), both for LBP and Hsp90 (S6 Fig). The 2D posterior *p*(*w*_open_, *d*_NC_|*D*, *K*) for LBP seemed converged at moderate computational effort of 50 ns per umbrella window (Fig 2D), whereas the 2D posterior $p(w_{\text{open}},R_{g}^{\text{open}}|D,K)$ for Hsp90 converged more slowly, as apparently from the somewhat scattered posteriors (Fig 4D). Hence, in future refinement simulations of very large systems such as the system of Hsp90, the sampling may benefit from additional enhanced sampling methods.

The computational cost of the simulations presented here are increased by only ∼15% as compared to standard MD simulations, suggesting that the calculations are well feasible on modern hardware. However, MD simulations are obviously more expensive than simplified methods such as rigid-body modelling.

The sampling of structural weights has some similarity with previous sample-and-select methods that reweighed a set of structures against SAXS data using, for instance, Bayesian or maximum-entropy criteria [16, 24–26]. However, at variance with previous methods, we refined the weights *simultaneously* with the structures, fitting parameters, and systematic errors. This difference is not a technical subtlety but is instead critical to estimate the correct uncertainty of the weights: In our method, by commitment to Bayesian inference, the uncertainty (or ignorance) about the structure, fitting parameters, and systematic errors are propagated into the uncertainty of the weights. In other words, when estimating the weights, and in contrast to previous methods, we do not assume any precise knowledge about the structure, fitting parameters, and systematic errors that, in truth, we do not have. This difference rationalizes why the uncertainties of fitted weights reported previously are much smaller than the uncertainties derived here via the full Bayesian treatment [24].

The refinement simulations presented here differ from previous methods for structure and ensemble refinement against SAXS data by a number of additional elements. First, since our refinement simulations are steered by the experimental SAXS data, the simulations are capable of sampling large-scale conformational transitions, which would not be sampled in an equilibrium simulation due to limited simulation time. An example is the open/close transition of Hsp90 that occurs on the time scale of many seconds at experimental conditions [49]. As such, our method does not strictly require the application of coarse-grained simulations [24, 25] or other simplified physical models [16, 26] to visit the relevant conformational states. Second, because we apply purely the MD force field and the SAXS data but no additional constraints, the refinement is not limited to rigid-body motions or normal modes, which were previously used to refine structures against SAXS data [14–17]. Hence, prior to the refinement simulations, our method does not require the ad-hoc definition of rigid bodies and flexible linkers, which may not be obvious. Third, in contrast to previous refinement methods, SAXS curves were computed using explicit-solvent algorithms, avoiding any solvent-related fitting parameters [21, 56].

In this study, we built upon the concept of “inferential structure determination” (ISD), which was originally formulated to model NMR data with a single structural state [5, 6]. In short, we presented an ISD method for SAXS data using all-atom MD simulations. In addition, we extended the ISD concept towards the refinement of a small number of states (typically two states), but the method is not intended for the refinement of continuous and highly heterogeneous ensembles. Hence, our approach complements methods for the reweighing of continuous ensembles against experimental data, as required for modeling of intrinsically disordered proteins [27, 57, 58], and it further complements maximum-entropy-based methods for biasing ensembles with experimental data [59–61].

We developed the method with a focus on SAXS data, but the calculations may be readily complemented by other sources of structural information. For instance, the refinement may be additionally guided by multiple sets of small-angle neutron scattering (SANS) data, optionally measured at various D_2_O contrasts and differently deuterated solutes. Similar to the SAXS-guided refinement, such SANS-guided refinement simulations will benefit from the fitting-free explicit-solvent scattering calculations applied here. Alternatively, the refinement simulations may be complemented by additional distance restraints from double electronelectron resonance (DEER) or FRET. Such future developments, complementing the method proposed here, may provide a route to MD-based Bayesian integrative modeling.

## Materials and methods

### Modeling of systematic errors

A common source of systematic errors in SAXS experiments is poor buffer matching. We therefore modeled the systematic errors *σ*_buf_ as a consequence of a buffer density mismatch *δρ*_buf_ between the protein solution and the pure buffer. Following previous work [18], *δρ*_buf_ can be translated into an uncertainty *σ*_buf_ of the calculated intensity *I*_c_(*q*), contributing to the likelihood function (see below).

### Likelihood function

We recently found excellent agreement of SAXS curves predicted from explicit-solvent MD simulations with experimental curves, if the experimental curves *I*_exp_(*q*) were adjusted by only two fitting parameters following *I*_fit_(*q*) = *fI*_exp_(*q*) + *c*, where *f* denotes the fitted absolute scale and *c* denotes a fitted constant offset [21], and *q* is the momentum transfer. Hence, we take for the likelihood function

$$L(D|R,w,\theta ,K)\propto \exp[-\frac{N_{\text{indep}}}{2N_{q}}\sum_{i=1}^{N_{q}}\frac{{\left[I_{\text{c}}\left(q_{i},R,w\right)-\left(fI_{\text{exp}}\left(q_{i}\right)+c\right)\right]}^{2}}{f^{2}\sigma_{\text{exp}}^{2}\left(q_{i}\right)+\sigma_{\text{calc}}^{2}\left(q_{i}\right)+\sigma_{\text{buf}}^{2}\left(q_{i};\delta \;\rho_{\text{buf}}\right)}],$$ (4)

where *θ* = {*f*, *c*, *δρ*_buf_}. As shown below, the fitting parameters *f* and *c* can be marginalized out analytically. The symbols *σ*_exp_ and *σ*_calc_ denote statistical errors of the experimental and calculated intensities, respectively. The calculated intensity *I*_*c*_ is a weighted average over the intensities of the *N* states, $I_{\text{c}}\left(q_{i},R,w\right)=\sum_{j=1}^{N}w_{j}I\left(q_{i},R_{j}\right)$. The symbols *N*_*q*_ and *N*_indep_ denote the total and the independent number of data points in the SAXS curve, respectively. *N*_indep_ was estimated by the number of Shannon-Nyqvist channels *N*_indep_ = *q*_max_ *D*_*p*_/*π*, where *D*_*p*_ is the maximum diameter of the protein and *q*_max_ is the maximum momentum transfer of the SAXS curve [13]. Hence, the factor *N*_indep_/*N*_*q*_ is an empirical correction that accounts for the fact that the number of independent data points *N*_indep_ in a SAXS curve is typically much smaller than the number of *q*-points *N*_*q*_ reported in experimental SAXS curves. Without the factor *N*_indep_/*N*_*q*_, the information content in the data would be overrated in comparison with the information in the priors. Critically, this correction assumes that the data *I*_exp_ corresponds to a “smoothed” SAXS curve, and that the experimental errors *σ*_exp_(*q*_*i*_) denote the true uncertainty of point *q*_*i*_ in the light of correlations of *I*_exp_ along *q*.

### Prior distributions

A flat prior was applied for the fitting parameters, *π*(*f*) = *π*(*c*) = 1. Notably, since the likelihood function is nonzero only for a very narrow *f*-range, applying the scale-invariant Jeffreys’ prior would change the posterior only marginally. The prior for the protein structure **R**_*j*_ of state *j* was taken from an unbiased MD simulation. Hence, *π*(**R**_*j*_|*K*) is given by a Boltzmann factor of the force field energy *V*_ff_, marginalized with respect to all solvent coordinates **r**_sol_ (water and ions), *π*(**R**_*i*_|*K*) ∝ ∫d**r**_sol_ exp[− *βV*_ff_(**R**_*i*_, **r**_sol_, *K*)]. Assuming no prior information on the weights, *π*(**w**|*K*) was taken as a flat Dirichlet distribution. For the buffer density mismatch *δρ*_buf_, a Gaussian prior was taken as $\pi \left(\delta \;\rho_{\text{buf}}\right)\propto \exp\left[-\delta \;\rho_{\text{buf}}^{2}/\left(2\epsilon_{\text{buf}}^{2}\right)\right]$. Here, *ϵ*_buf_ is a free parameter that quantifies the uncertainty of the density of an experimental buffer. Typical values for *ϵ*_buf_ would be 0.1 to 0.5% of the density of water, yet we found that the choice for *ϵ*_buf_ had only a small effect on *p*(**R**, **w**).

### On-the-fly calculation of SAXS curves from MD simulations

The buffer-subtracted SAXS curves were derived by explicit-solvent calculations, as described previously [18, 21]. Because the explicit solvent provides an accurate model for the hydration layer and excluded solvent, these calculations did not require any solvent-related fitting parameters, in contrast to implicit-solvent SAXS calculations.

In short, a spatial envelope was constructed around the protein at a distance of at least 8Å from all protein atoms (Fig 1). All protein and solvent atoms within the envelope were taken into account for the calculation of the SAXS curve, as visualized in Fig 1. Likewise, scattering contributions from the excluded solvent were computed from solvent atoms within the envelope taken from a pure-water MD simulation. A memory time constant of *τ* = 500 ps was applied during both LBP and Hsp90 simulations. The orientational average (or spherical quadrature) was conducted numerically using 1200 **q**-vectors per absolute value of *q*, distributed by the spiral method. During SAXS refinement simulations, the SAXS curves were updated on-the-fly every 0.5 ps. The statistical uncertainty *σ*_c_ of the calculated intensity was computed by applying standard Gaussian error propagation to the SAXS intensity calculations we described previously [21]. After averaging over a few hundred MD frames, *σ*_c_ is typically small compared to the other uncertainties that contribute to the likelihood function (*σ*_buf_ and *σ*_exp_).

The SAXS curves of the purely open and purely closed states of LBP (Fig 2, solid lines) were computed from 100-nanosecond free simulations of the open and closed state.

### Marginalizing out the fitting parameters *f* and *c*

The two fitting parameters *f*, corresponding to the absolute scale of the SAXS curve, and the offset *c*, can be marginalized out analytically at the level of the likelihood. Assuming Gaussian errors, we take for the likelihood

$$L(D|R,w,f,c,\;\delta \rho_{\text{buf}},K)\propto \exp\left[-\frac{1}{2}\zeta \chi^{2}\right]$$ (5)

with

$$\begin{array}{c} \chi^{2}=\sum_{i=1}^{N_{q}}\tau_{i,f}{\left[I_{\text{c}}\left(q_{i},R,w\right)-\left(fI_{\text{exp}}\left(q_{i}\right)+c\right)\right]}^{2}, \end{array}$$ (6)

where we introduced the symbol *ζ* = *N*_indep_/*N*_*q*_, as well as the precision of the *i*^th^ *q*-point as follows:

$$\begin{array}{c} \tau_{i,f}=\frac{1}{\sigma^{2}\left(q_{i}\right)}=\frac{1}{f^{2}\sigma_{\text{exp}}^{2}\left(q_{i}\right)+\sigma_{\text{c}}^{2}\left(q_{i}\right)+\sigma_{\text{buf}}^{2}\left(q_{i};\delta \;\rho_{\text{buf}}\right)}. \end{array}$$ (7)

Here, we used that the uncertainties from the experiment *σ*_exp_, from the calculation *σ*_c_, and from the buffer subtraction *σ*_buf_ are independent, suggesting that the respective variances add up to the total variance *σ*^2^(*q*_*i*_). The precision *τ*_*i*,*f*_ depends on the fitted scale *f* because the experimental errors *σ*_exp_ must be scaled simultaneously with the experimental intensities *I*_exp_. To allow us to marginalize out the scale *f* analytically, we use that the errors in the small-angle regime are much smaller than the intensities, suggesting that purely values of *f* close to it’s maximum-likelihood estimate *f*_ml_ contribute to the marginalized likelihood. As a consequence, replacing *f* by *f*_ml_ in eq 7 has only a small effect on the marginalized likelihood. We therefore use for the precision in the following

$$\begin{array}{c} \tau_{i}={\left[f_{\text{ml}}^{2}\sigma_{\text{exp}}^{2}\left(q_{i}\right)+\sigma_{\text{calc}}^{2}\left(q_{i}\right)+\sigma_{\text{buf}}^{2}\left(q_{i};\delta \;\rho_{\text{buf}}\right)\right]}^{-1}. \end{array}$$ (8)

In the first calculation step, while *f*_ml_ is still unknown, it may be simply estimated from the non-weighted averages of the calculated and experimental intensities, following *f*_ml_ ≈ ∑_*i*_ *I*_*c*_(*q*_*i*_)/∑_*i*_ *I*_exp_(*q*_*i*_).

To keep the nomenclature clear, let us introduce additional symbols. Let $T:=\sum_{i=1}^{N_{q}}\tau_{i}$ denote the sum over all precisions. The *τ*-weighted average over *q*-points is

$$\begin{array}{c} \left\langle X\right\rangle =T^{-1}\sum_{i=1}^{N_{q}}\tau_{i}X\left(q_{i}\right). \end{array}$$ (9)

With the last definition, the *τ*-weighted variances of the calculated and experimental SAXS intensities are

$$\begin{array}{ccc} s_{c}^{2} & = & \left\langle I_{c}^{2}\right\rangle -{\left\langle I_{c}\right\rangle }^{2} \end{array}$$ (10)

$$\begin{array}{ccc} s_{\text{exp}}^{2} & = & \left\langle I_{\text{exp}}^{2}\right\rangle -{\left\langle I_{\text{exp}}\right\rangle }^{2}, \end{array}$$ (11)

respectively, and the *τ*-weighted Pearson correlation coefficient between the calculated and experimental data points is

$$\begin{array}{c} P=\frac{\left\langle I_{c}\;I_{\text{exp}}\right\rangle -\left\langle I_{c}\right\rangle \left\langle I_{\text{exp}}\right\rangle }{s_{c}\;s_{\text{exp}}}. \end{array}$$ (12)

The maximum likelihood estimates for the fitting parameters *f* and *c* are

$$\begin{array}{ccc} f_{\text{ml}} & = & P\frac{s_{c}}{s_{\text{exp}}} \end{array}$$ (13)

$$\begin{array}{ccc} c_{\text{ml}} & = & \left\langle I_{c}\right\rangle -f_{\text{ml}}\left\langle I_{\text{exp}}\right\rangle , \end{array}$$ (14)

respectively. The residual between *I*_*c*_ and *I*_exp_ that cannot be fitted by the parameters *f* and *c* is

$$\begin{array}{ccc} {\hat{\chi }}^{2} & = & T[s_{c}^{2}-{\left(f_{\text{ml}}s_{\text{exp}}\right)}^{2}] \end{array}$$ (15)

$$\begin{array}{ccc} & = & T\langle {\left[I_{c}-\left(f_{\text{ml}}I_{\text{exp}}+c_{\text{ml}}\right)\right]}^{2}\rangle . \end{array}$$ (16)

The last equality is derived using eqs 10 to 14. The likelihood *L*_marg_ marginalized with respect to fitting parameters *f* and *c* is obtained by integrating over *f* and *c*. Since no prior information on *f* and *c* is available, we assumed flat prior distributions, *π*(*f*) = *π*(*c*) = 1. A straightforward calculation yields:

$$\begin{array}{c} \begin{array}{cc} L_{\text{marg}} & \left(D|R,w,\sigma_{\text{buf}},K\right)\\ & \propto \int df\int dc\;L\left(D|R,w,f,c,\sigma_{\text{buf}},K\right)\pi \left(f\right)\pi \left(c\right)\\ & \propto \frac{1}{Ts_{\text{exp}}}\exp(-\frac{1}{2}\zeta {\hat{\chi }}^{2}) \end{array} \end{array}$$ (17)

Here, we dropped the normalization factors and other constants of the likelihood because these only lead to an irrelevant constant offset in the experiment-derived energies.

### Force calculations

In order to sample the posterior distribution using Newtonian dynamics, *L*_marg_ is reformulated as its energy analogue

$$\begin{array}{c} E_{\text{exp}}=-\beta^{-1}\ln L_{\text{marg}}. \end{array}$$ (18)

Using eqs 10 through 18, the experiment-derived force on atom *ℓ* of state *j* to is

$$\begin{array}{ccc} F_{j,\ell } & = & -\frac{\partial }{\partial r_{j,\ell }}E_{\text{exp}} \end{array}$$ (19)

$$\begin{array}{ccc} & = & -\beta^{-1}\zeta \sum_{i=1}^{N_{q}}\tau_{i}[I_{c}\left(q_{i}\right)-\left(f_{\text{ml}}I_{\text{exp}}\left(q_{i}\right)+c_{\text{ml}}\right)]\frac{\partial I_{c}\left(q_{i},R,w\right)}{\partial r_{j,\ell }}, \end{array}$$ (20)

where **r**_*j*,*ℓ*_ denotes the Cartesian coordinates of atom *ℓ* in state *j* (*j* = 1, …, *N*). In general, the calculated SAXS intensity *I*_*c*_ is a weighted average over the intensities of the *N* states:

$$\begin{array}{c} I_{c}\left(q_{i},R,w\right)=\sum_{j=1}^{N}w_{j}I\left(q_{i},R_{j}\right), \end{array}$$ (21)

where *w*_*j*_ and *I*(*q*_*i*_, **R**_*j*_) denote the normalized weight (∑_*j*_ *w*_*j*_ = 1) and the SAXS intensity of state *j*, respectively. Following eq 21, the derivative of *I*_*c*_ with respect to **r**_*j*,*ℓ*_ depends purely on the SAXS intensity of state *j*:

$$\begin{array}{c} \frac{\partial I_{c}\left(q_{i},R,w\right)}{\partial r_{j,\ell }}=w_{j}\frac{\partial I(q_{i},R_{j})}{\partial r_{j,\ell }}. \end{array}$$ (22)

Note that, for the simulations conducted in this study, one closed state (*j* = 1) was assumed to adopt a fixed know structure, whereas a second open state (*j* = 2) was refined against SAXS data. Hence, the forces **F**_*j*,*ℓ*_ were purely evaluated for the second flexible state. However, the equations presented above are suitable for simultaneously refining multiple states against SAXS data. The derivative ∂*I*(*q*_*i*_, **R**_*j*_)/∂**r**_*j*,*ℓ*_ was computed as described previously [18, 21].

### Marginalizing out the scale *f* only

For the simulations of this study, we applied the likelihood function defined in eqs 5 and 6, using both the absolute scale *f* and the constant offset *c* as unknown fitting parameters. However, there may be applications for which the fitting of a constant offset *c* is undesirable. Hence, for the sake of completeness, we report the expressions for marginalizing out purely the absolute scale *f*.

Then, the likelihood takes the form of eqs 5 and 6 with the parameter *c* set to zero. The maximum-likelihood estimate for the scale *f* evaluates to $f_{\text{ml}}^{\prime }=\langle I_{c}\;I_{\text{exp}}\rangle /\langle I_{\text{exp}}^{2}\rangle$, and the residual between *I*_*c*_ and *I*_exp_ changes to

$$\begin{array}{c} {\hat{\chi^{\prime }}}^{2}=T\left(\left\langle I_{c}^{2}\right\rangle -{\left\langle I_{c}I_{\text{exp}}\right\rangle }^{2}/\left\langle I_{\text{exp}}^{2}\right\rangle \right)=T\left\langle {\left[I_{c}-f_{\text{ml}}^{\prime }I_{\text{exp}}\right]}^{2}\right\rangle . \end{array}$$ (23)

The marginalized likelihood is

$$\begin{array}{c} L_{\text{marg}}^{\prime }\propto \frac{1}{{\left[T\left\langle I_{\text{exp}}\right\rangle \right]}^{1/2}}\exp(-\frac{1}{2}\zeta {\hat{\chi^{\prime }}}^{2}), \end{array}$$ (24)

and the force on atom *ℓ* of state *j*

$$\begin{array}{c} F_{j,\ell }=-\beta^{-1}\zeta \sum_{i=1}^{N_{q}}\tau_{i}[I_{c}\left(q_{i}\right)-f_{\text{ml}}I_{\text{exp}}\left(q_{i}\right)]\frac{\partial I_{c}\left(q_{i}\right)}{\partial r_{j,\ell }}. \end{array}$$ (25)

### Monte-Carlo moves for *δρ*_buf_ and weights

The weights of the *N* states (*N* = 2 in this study), as well as the uncertainty of the buffer density *δρ*_buf_ were sampled using Gibbs sampling, that is, using Monte-Carlo (MC) moves with all other parameters fixed. At each time step at which the SAXS intensities were updated (0.5 ps in this study), 20 rounds of MC moves of *δρ*_buf_ and *w*_open_ were conducted. In each round, 20 MC moves of *δρ*_buf_ were conducted (at fixed *w*_open_), followed by 20 MC moves of *w*_open_ (at fixed *δρ*_buf_). Typical posteriors of the parameter *δρ*_buf_ are shown in S3 Fig.

Proposed MC moves of *δρ*_buf_ were taken from a uniform distribution in the interval [0, 6*ϵ*_buf_). Proposed MC moves for the weight vector **w** = (*w*_1_, …, *w*_*N*_) were taken from a flat Dirichlet distribution. Hence, proposed **w** satisfied $\sum_{i=1}^{N}w_{i}=1$ and were uniformly distributed over the standard (*N* − 1)-simplex, that is, the prior *π*(**w**) was a constant. Such **w** were drawn from the flat Dirichlet distribution by randomly partitioning the interval [0, 1], as follows:

1. Take random numbers *R*_*i*_ (*i* = 1, …, *N* − 1) from a uniform distribution in the interval [0, 1]. In addition, set *R*_0_ = 0 and *R*_*N*_ = 1.
2. Sort the *R*_*i*_ in increasing order.
3. Take proposed weights as *w*_*i*_ = *R*_*i*_ − *R*_*i* − 1_.

We noticed that restricting the sampling of *w*_*i*_ to the interval [0, 1] may lead to artifacts at “edge” of the (*N* − 1)-simplex, presumably as a consequence of the weighted running averages used for computing SAXS curves on-the-fly during MD simulations [18]. To avoid a boundary in the physically relevant weights space, the sampled weight space was extended to unphysical but mathematically well-defined weights slightly outside the (*N* − 1)-simplex (outside the interval [0, 1] in case of *N* = 2). This was achieved by scaling the proposed weight vector **w**, followed by a shift along the vector with all elements equal to unity, **j** = (1, …, 1), as follows:

$$\begin{array}{c} w^{\prime }=\left(1+\xi N\right)w-\xi \;j. \end{array}$$ (26)

The parameter *ξ* was set to 0.1 in this study. This transformation keeps the prior of **w**′ uniform on the (*N* − 1)-simplex, and it keeps the weight vector normalized ($\sum_{i=1}^{N}w_{i}^{\prime }=1$). However, it allows one to draw samples of $w_{i}^{\prime }$ from the interval [−*ξ*, 1 + *Nξ* − *ξ*]. For *N* = 2, for instance, samples of $w_{i}^{\prime }$ are drawn from the interval [−*ξ*, 1 + *ξ*].

The proposed MC move was accepted with probability *P*_accept_ according to the Metropolis algorithm,

$$\begin{array}{c} P_{\text{accept}}=\text{min}\left\{1,p_{\text{marg}}^{\prime }/p_{\text{marg}}\right\}, \end{array}$$ (27)

where the prime indicates the posterior after the MC move. Further, the symbol *p*_marg_ denotes the posterior distribution after marginalizing out the fitting parameters, which is given by

$$p_{\text{marg}}(R,w,\delta \rho_{\text{buf}}|D,K)\propto L_{\text{marg}}(D|R,w,\delta \rho_{\text{buf}},K)\pi (R|K)\pi (w|K)\pi (\delta \rho_{\text{buf}}|K).$$ (28)

For each MC move *p*_marg_ was evaluated using eq 17 as well as the priors for **w** (a constant in this study) and *π*(*σ*_buf_) (a Gaussian in this study, see section on prior distributions).

### Umbrella sampling along open/closed weights

Obtaining a (reasonably) converged posterior distribution as a function of weights and protein coordinates would require very long simulations. To ensure exhaustive sampling of the weights space and, hence, to accelerate the convergence of the posterior, we used umbrella sampling along the weights [32]. Further, umbrella sampling is technically convenient because it allows the calculation of the posterior from a set of independent simulations.

For the two-state refinement used here, one-dimensional umbrella sampling was sufficient. Accordingly, the weight of the open state *w*_open_ was decomposed into *N*_win_ = 11 umbrella windows $w_{\text{open}}^{\left(k\right)}=\left\{0,0.1,\ldots ,1.0\right\}$ (*k* = 1, …, *N*_win_). During MC moves of the weights, a harmonic umbrella potential was applied $V_{k}^{\left(b\right)}=f_{w}{\left(w_{\text{open}}-w_{\text{open}}^{\left(k\right)}\right)}^{2}/2$ or, equivalently, the MC moves were accepted or rejected based on the biased posterior

$$\begin{array}{c} p_{\text{marg},k}^{\left(b\right)}=p_{\text{marg}}\;e^{-\beta V_{k}^{\left(b\right)}}. \end{array}$$ (29)

An umbrella force constant of *f*_*w*_ = 1000 kJ/mol was applied. A typical set of umbrella histograms is shown in S4 Fig, demonstrating sufficient overlap between neighboring histograms. After the simulations had finished, the umbrella windows were combined to the unbiased posterior using the weighted histogram analysis method (WHAM), as implemented in the g_wham software [62, 63].

### Schematic overview of the algorithm

**Fig 7:**
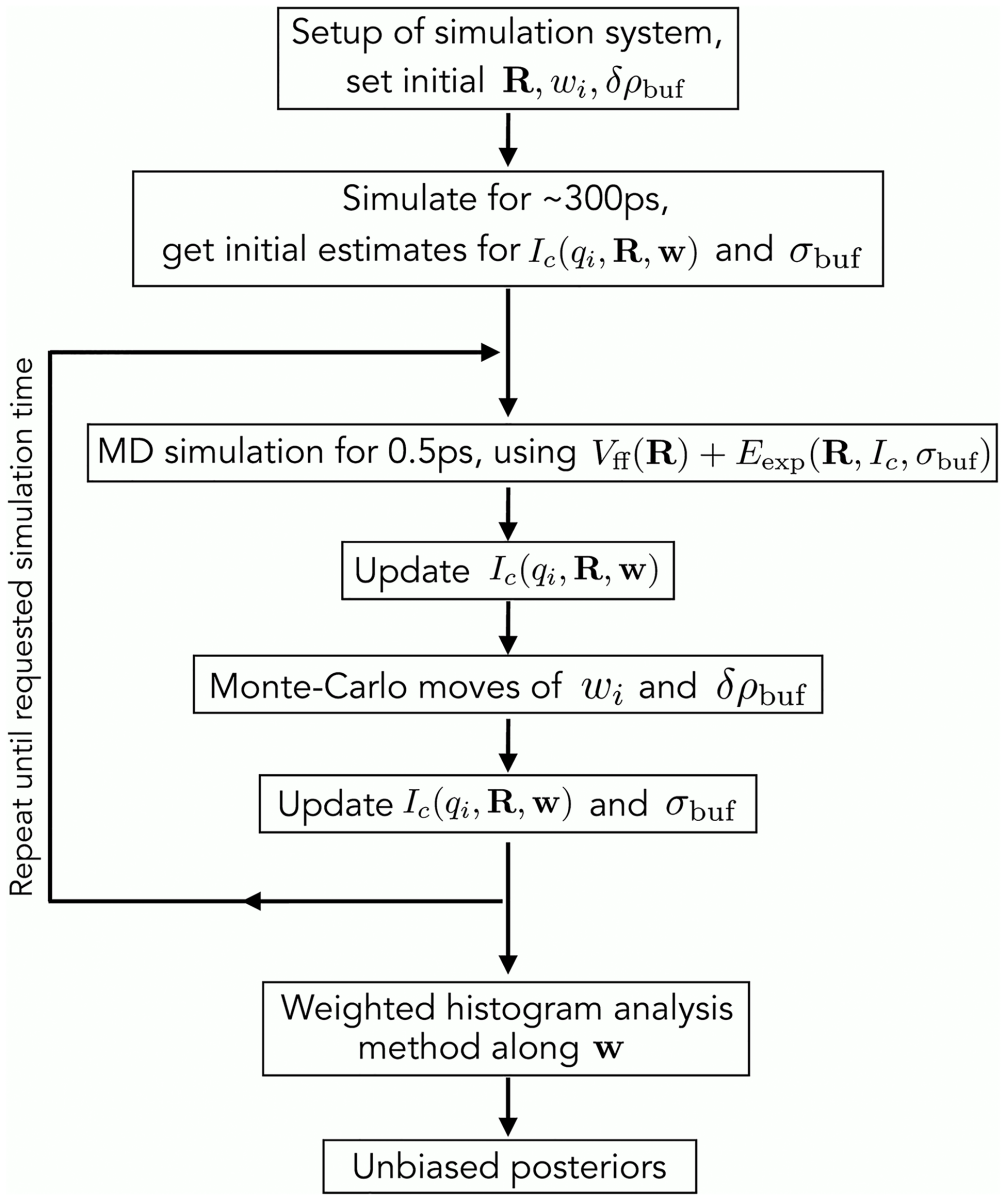
Overview of the algorithm used to compute the posteriors. For more details, see text.

Fig 7 visualizes the algorithm used to compute the posteriors. Accordingly, the simulation system is set up from the initial coordinates **R**, and initial values for the weights **w** and the buffer density mismatch *δρ*_buf_ are defined. The system is freely simulated for *τ* (the memory time constant for on-the-fly SAXS calculations [18]), using purely the MD force field *V*_ff_. The free simulation is required is required because, using the explicit-solvent SAXS predictions, the SAXS curve cannot be computed from a single frame but instead requires averaging over solvent fluctuations. Within the free simulation, an initial estimate for the calculated SAXS intensity *I*_*c*_(*q*_*i*_, **R**, **w**) is obtained. A typical value for *τ* is 300 ps, suggesting that the computed SAXS curves account for fluctuations on the several hundred picosecond time scale. Subsequently, the experiment-derived energy *E*_exp_ is gradually turned on within the following *τ*.

The following steps are repeated until the requested simulation time is reached for each umbrella window along the weights: (i) MD simulation using forces derived from the hybrid energy, i.e., using the potential *V*_ff_ + *E*_exp_; (ii) update of *I*_*c*_(*q*_*i*_, **R**, **w**) based on the current MD frame and using a cumulative weighted average [18], as previously suggested for NMR refinement [64]; (iii) a few hundred MC moves of weights *w*_*i*_ and *δρ*_buf_ (see above); (iv) update *I*_*c*_(*q*_*i*_, **R**, **w**) with the final weights, and update the systematic error *σ*_buf_ with the final *δρ*_buf_, as described previously [18]. After the simulations from all umbrella windows have finished, the biased posteriors from all windows are combined into the unbiased posterior using WHAM [62].

### Preparation of structures for MD simulations

The crystal structures of the apo and holo states of LBP were taken from the protein data bank (PDB codes 1USG and 1USI [35]). For the simulation of Hsp90 the structure of ATP-bound yeast Hsp90 was used (PDB code 2CG9) [36]. The co-chaperone proteins SBA1 and ATP ligands were removed from the structure of HspP90 and leucine ligand was removed from the LBP structure. Flexible linkers missing in the Hsp90 crystal structure were added using Modeller [65]. A swap of the N-terminal *β*-strand (residues 1-9), which prevented the opening of the protein, was removed using the Coot software [66].

The structures were placed in simulation boxes of a rhombic dodecahedron with distance between the protein and the box surface of 1.5 and 4 nm for LBP and Hsp90, respectively. The systems were solvated by explicit water. Sodium and chloride ions were added to obtain a salt concentration of 100 mM. Here, the number of sodium and chloride ions was adjusted to neutralize the system. The energies of the systems were minimized using the steepest descent algorithm for 2000 steps. Subsequently, the systems were equilibrated with position restraints on the backbone atoms for 10 and 20 ns for LBP and Hsp90, respectively.

To obtain an initial open structure of Hsp90, we carried out pulling simulations along the distance of the two N-terminal domains. Accordingly, the center-of-mass distance between the two N-terminal domains was increased from 4 nm to 8 nm within 40 ns, using a pulling speed of 0.1 nm/ns. The obtained open structure was resolvated in a larger simulation box with the distance between the protein and the box surface of 3 nm, and the structure was equilibrated for another 20 ns with position restraints on the backbone atoms. The final structure was used as a starting structure for SAXS refinement. The Hsp90 system contained approximately 1.5 × 10^6^ atoms.

### MD parameters

Standard MD simulations were performed using the GROMACS simulation software (version 4.6) [67]. All SAXS calculations were done with an in-house modification of GROMACS 4.6, which is available from the authors upon request. Protein interactions of LBP and Hsp90 were taken from the CHARMM27 and CHARMM22* forcefields, respectively [68, 69], and water was modeled by the TIP3P potential [70]. Hydrogen atoms of the proteins were modeled as virtual interaction sites allowing an integration timestep of 4 fs. Electrostatic interactions were treated with the particle-mesh Ewald scheme [71, 72]. The cutoff of 1.2 nm was applied to the direct-space Coulomb and Lennard-Jones interactions. The bond lengths and angles of water molecules were constrained with the SETTLE algorithm [73], and all other bonds were constrained with LINCS [74]. The pressure was set to 1 bar using the Berendsen barostat (*τ* = 1 ps) [75]. During equilibration runs, the temperature was controlled at 300 K with the Berendsen thermostat (*τ* = 0.5 ps) [75]. During SAXS-driven simulations, in contrast, a tight stochastic dynamics integration scheme was applied, motivated from the fact that SAXS-driven MD is not strictly energy conservative [76].

### SAXS refinement simulations

For LBP simulations, the target curves for the refinement were modeled from calculated SAXS curves of the closed state *I*_closed_(*q*) (Fig 2C, solid dark blue curve) and open state *I*_open_(*q*) (Fig 2C, solid light blue curve), as follows:

$$\begin{array}{c} I_{\text{exp},w}\left(q\right)=w_{\text{open}}^{\text{exp}}I_{\text{open}}\left(q\right)+\left(1-w_{\text{open}}^{\text{exp}}\right)I_{\text{closed}}\left(q\right) \end{array}$$ (30)

In this study, we tested ensemble refinement against SAXS data computed with the following $w_{\text{open}}^{\text{exp}}$: 0, 25%, 50%, 75%, or 100% (Fig 2C, solid and dashed curves). Hence, since *I*_exp,*w*_(*q*) was computed theoretically, the true weight of the open state was known, allowing us

1. to validate that the SAXS-guided refinement starting from the closed state is capable of reproducing the true weight ($w_{\text{open}}^{\text{exp}}$) and the true structure of the open state (used to compute *I*_open_(*q*)), and
2. to derive the uncertainty (or ambiguity) of the weight and structure in the light of the SAXS curve and the MD force field, as given by the width of the posterior distributions.

All simulations of LBP were started from the closed state (Fig 2A). The simulations were coupled to the target SAXS curve at *N*_*q*_ = 25 *q*-points, which were evenly distributed between 0 and 8 nm^−1^. The two-state ensemble refinement was conducted using umbrella sampling along the weight *w*_open_ of the open state (see above). Each umbrella window was simulated for 40 ns, where the first 2 ns were removed for equilibration. The posterior distributions of *w*_open_ and of the interdomain distance derived from these simulations are presented in Figs 2D, 3A and S1A Fig.

For comparison, a single state (instead of the ensemble of two states) was refined against each of the five curves *I*_exp,*w*_(*q*), using five simulations of 10 ns each and removing the first 2 ns for equilibration. S1B Fig presents the posteriors of the interdomain distance *d*_NC_ resulting from refining a single structure against SAXS curves that, in truth, represent heterogenous open/closed ensemble. Notably, the single-state refinements try to explain those SAXS curves with intermediate (partially open) structures.

For the refinement simulations of Hsp90, the simulations were coupled to the target SAXS curve at *N*_*q*_ = 30 *q*-points, which were evenly distributed between 0.1 and 3 nm^−1^. The *q*-range below 0.1 nm^−1^ was omitted because the experimental data exhibited some deviation from the ideal Guinier behaviour. For some umbrella windows, Hsp90 was required to carry out large-scale conformational transitions. To accelerate those transitions, each window was first simulated for 8 ns with a ten-fold increased experiment-derived energy *E*_exp_. Subsequently, the simulation of each umbrella window was continued for another 20 ns using the statistically founded *E*_exp_ that leads to the correct posterior (eq 18). From those simulations, the first 2 ns were removed for further equilibration, and the remaining simulation time was used to compute the posterior. An example of the umbrella histograms along the weight coordinate is shown in S4 Fig. To further improve the sampling close to the maxima of the posteriors, the simulations of the umbrella window at the peak of *p*(*w*_open_|*D*, *K*) plus two neighboring windows were prolonged for another 15 ns.

## Supporting information

S1 Fig Marginalized posteriors of the interdomain distance *d*_NC_ of the refined open state, taken from two-state and single-state refinement simulations of LBP. (A) Marginalized posteriors of the interdomain distance *d*_NC_ of the refined open state, taken from two-state refinement simulations of LBP. In ensembles refined against SAXS curves of non-zero open-state content (25:75 through 100:0), the posteriors peak near the physically correct *d*_NC_ of ∼3.25 nm of the open state. In the ensemble refined against the SAXS curve of purely the closed state (0:100), the refined weight of the open state is near zero (Fig 3A of main text), suggesting that the simulation of the open state is hardly restrained by the SAXS curve or, equivalently, is essentially a free simulation. Consequently, the posterior of *d*_NC_ (A, dark green) is wide and reflects both closed and open states.
(PDF)

S2 Fig Target SAXS curves (black lines) and calculated SAXS curves of the refined structures and ensembles (red dots) of leucine binding protein. (A/C) Two-state ensemble refinement. (B/D) Single-state refinement. SAXS curves with open weight ≥25% were offset for clarity. The lower row (C/D) shows a close-up view on the small-angle regime.
(PDF)

S3 Fig Posterior of *δρ*_buf_/*ϵ*_buf_ during two state refinement of LBP and Hsp90 refinement. For LBP, the ensembles were refined against theoretically computed SAXS curves, thus exhibiting no buffer density mismatch, rationalizing why the posteriors peak near *δρ*_buf_ = 0. For Hsp90, in contrast, the ensembles were refined against experimental data that presumably exhibit some systematic errors, for instance due to a small buffer density mismatch. Hence, the posteriors peak at nonzero *δρ*_buf_.
(PDF)

S4 Fig Example of umbrella histograms. Umbrella histograms along the weight of the open state, here taken from the two-state refinement of LBP against the open/closed 50:50 SAXS curve.
(PDF)

S5 Fig Root mean-square deviation (RMSD) analysis of LBP. Root mean-square deviation (RMSDs) between the C_*α*_ atoms of (i) the refined open structure of LBP and (ii) the open LBP structure (similar to Fig 2B). For the RMSD calculations, the open structure was taken as the average structure of the ensembles used to compute the SAXS curve of the open state. Trajectories were taken from umbrella windows of *w*_open_ close to the peak of the posterior *p*(*w*_open_|*D*, *K*). The color indicates the open:closed weights used to compute the target curve (see legend, five target curves in Fig 2C). The RMSD curves demonstrate that, starting from the closed state, LBP rapidly opens and approaches the open structure. Some RMSD fluctuations after longer time (green curves) reflect a smaller twist motion between the N- and C-terminal domains within the open state. Such fluctuations along the twist are expected since, as shown previously [18], the SAXS data restrains the degree of openness but not the twist.
(PDF)

S6 Fig Analysis of the convergence of posterior distributions with increasing invested simulation time. (A/B) Marginalized posteriors for LBP refined against the SAXS curve with 50:50 open/closed weight (Fig 2D, middle panel), computed from time bins as indicated in the legend. (A) Posterior of the interdomain distance *d*_NC_ and (B) of the weight of the open state *w*_open_. (C) Marginalized posteriors of *w*_open_ for different liganded states of Hsp90 as indicated in the legend. The thin lines indicate posteriors computed from an increasing number of histograms: 10 equally spaced histograms, the same 10 plus additional three histograms near the posterior maximum, 10 plus 6 additional histograms, and 10 plus 9 additional histograms near the posterior maximum. The similarity between the posteriors suggest that the posteriors are reasonably converged.
(PDF)

S1 Table Maxima and confidence intervals of *w*_*open*_, taken from *p*(*w*_*open*_|*D*, *K*) of the two-state ensemble refinement of LBP. All numbers in %. The respective posteriors are shown in Fig 3A.
(PDF)

S2 Table Maxima and confidence intervals of *d*_*NC*_, taken from *p*(*d*_*NC*_|*D*, *K*) of the two-state ensemble refinement of LBP. During two-state refinement, in simulations with non-zero *w*_open_, the posterior of the interdomain distance *p*(*d*_*NC*_|*D*, *K*) of the open state peaks at the physically correct *d*_*NC*_ ≈ 3.25 nm. The respective posteriors are shown in S1A Fig. All distances are in nanometers.
(PDF)

S3 TableMaxima and confidence intervals of *d*_*NC*_ taken from *p*(*d*_*NC*_|*D*, *K*) of the single-state refinement of LBP.Refining a single state against SAXS curves that, in truth, represent a heterogenous ensemble of open/closed states, yields posterior distributions that peak at the “mean” interdomain distance $\langle d_{\text{NC}}\rangle =w_{\text{open}}d_{\text{NC}}^{\text{open}}+\left(1-w_{\text{open}}\right)d_{\text{NC}}^{\text{closed}}$, where $d_{\text{NC}}^{\text{open}}$ and $d_{\text{NC}}^{\text{closed}}$ denote the mean interdomain distances of the open and closed states, in free simulations, respectively. The respective posteriors are shown in Fig 1B. All distances are in nanometers.(PDF)

S4 Table Maxima and confidence intervals of *w*_*open*_ taken from *p*(*w*_*open*_|*D*, *K*) of the two-state ensemble refinement of Hsp90. All numbers in %. The respective posteriors are shown in Fig 4D of the main text.
(PDF)

S1 Archive Source code of modified version of gromacs which was used for SAXS-driven MD simulations. (BZ2)

S2 Archive Setup files for the LBP two-state ensemble refinement. (BZ)

S3 Archive Setup files for the HSP90 two-state ensemble refinement. (BZ)
