# Supplementary material for: Bayesian refinement of protein structures and ensembles against SAXS data using molecular dynamics
Source: PLoS Comput Biol. 2017 Oct 18;13(10):e1005800. doi: 10.1371/journal.pcbi.1005800 (PMC5662244; doi:10.1371/journal.pcbi.1005800)
Supplement: S1 Fig — (A) Marginalized posteriors of the interdomain distance dNC of the refined open state, taken from two-state refinement simulations of LBP. In ensembles refined against SAXS curves of non-zero open-state content (25:75 through 100:0), the posteriors peak near the physically correct dNC of ∼3.25 nm of the open state. In the ensemble refined against the SAXS curve of purely the closed state (0:100), the refined weight of the open state is near zero (Fig 3A of main text), suggesting that the simulation of the open state is hardly restrained by the SAXS curve or, equivalently, is essentially a free simulation. Consequently, the posterior of dNC (A, dark green) is wide and reflects both closed and open states. (PDF) [file pcbi.1005800.s001.pdf]

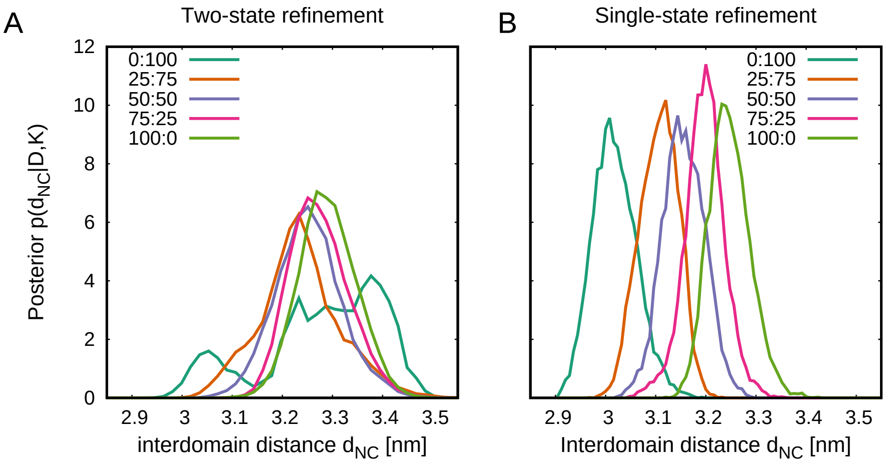

Figure S1. Marginalized posteriors of the interdomain distance d_{NC} of the refined open state, taken from two-state and single-state refinement simulations of LBP. (A) Marginalized posteriors of the interdomain distance d_{NC} of the refined open state, taken from two-state refinement simulations of LBP. In ensembles refined against SAXS curves of non-zero open-state content (25:75 through 100:0), the posteriors peak near the physically correct d_{NC} of $\sim$3.25 nm of the open state. In the ensemble refined against the SAXS curve of purely the closed state (0:100), the refined weight of the open state is near zero (Fig. 3A of main text), suggesting that the simulation of the open state is hardly restrained by the SAXS curve or, equivalently, is essentially a free simulation. Consequently, the posterior of d_{NC} (A, dark green) is wide and reflects both closed and open states.
