# Supplementary material for: Bayesian refinement of protein structures and ensembles against SAXS data using molecular dynamics
Source: PLoS Comput Biol. 2017 Oct 18;13(10):e1005800. doi: 10.1371/journal.pcbi.1005800 (PMC5662244; doi:10.1371/journal.pcbi.1005800)
Supplement: S2 Fig — (A/C) Two-state ensemble refinement. (B/D) Single-state refinement. SAXS curves with open weight ≥25% were offset for clarity. The lower row (C/D) shows a close-up view on the small-angle regime. (PDF) [file pcbi.1005800.s002.pdf]

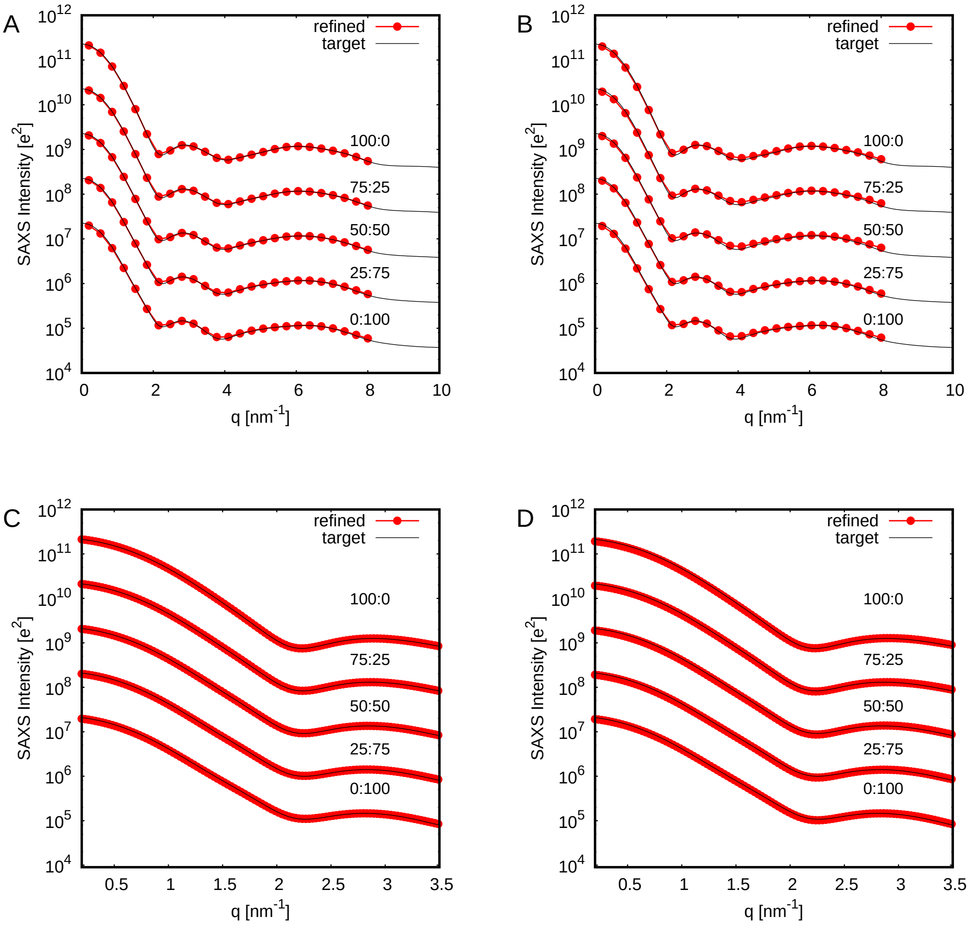

Figure S2. Target SAXS curves (black lines) and calculated SAXS curves of the refined structures and ensembles (red dots) of leucine binding protein. (A/C) Two-state ensemble refinement. (B/D) Single-state refinement. SAXS curves with open weight ≥25% were offset for clarity. The lower row (C/D) shows a close-up view on the small-angle regime.
