# Supplementary material for: Bayesian refinement of protein structures and ensembles against SAXS data using molecular dynamics
Source: PLoS Comput Biol. 2017 Oct 18;13(10):e1005800. doi: 10.1371/journal.pcbi.1005800 (PMC5662244; doi:10.1371/journal.pcbi.1005800)
Supplement: S3 Fig — For LBP, the ensembles were refined against theoretically computed SAXS curves, thus exhibiting no buffer density mismatch, rationalizing why the posteriors peak near δρbuf = 0. For Hsp90, in contrast, the ensembles were refined against experimental data that presumably exhibit some systematic errors, for instance due to a small buffer density mismatch. Hence, the posteriors peak at nonzero δρbuf. (PDF) [file pcbi.1005800.s003.pdf]

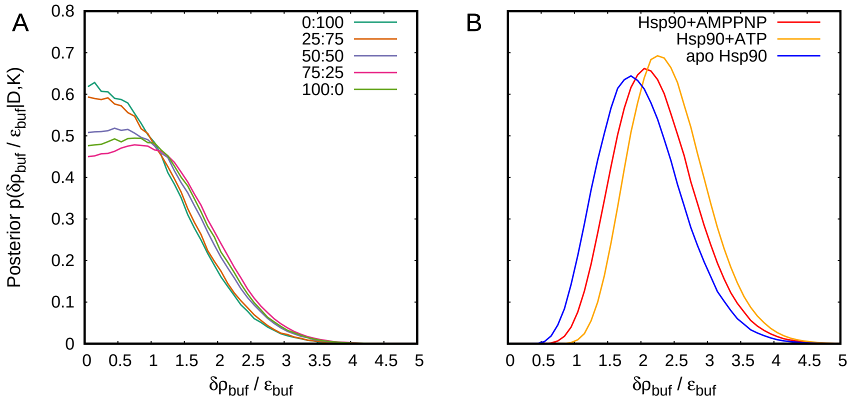

Figure S3. Posterior of $\delta\rho_{\mathbf{buf}}/\epsilon_{\mathbf{buf}}$ during two state refinement of LBP and Hsp90 refinement. For LBP, the ensembles were refined against theoretically computed SAXS curves, thus exhibiting no buffer density mismatch, rationalizing why the posteriors peak near $\delta\rho_{\mathrm{buf}} = 0$. For Hsp90, in contrast, the ensembles were refined against experimental data that presumably exhibit some systematic errors, for instance due to a small buffer density mismatch. Hence, the posteriors peak at nonzero $\delta\rho_{\mathrm{buf}}$.
