# Supplementary material for: Bayesian refinement of protein structures and ensembles against SAXS data using molecular dynamics
Source: PLoS Comput Biol. 2017 Oct 18;13(10):e1005800. doi: 10.1371/journal.pcbi.1005800 (PMC5662244; doi:10.1371/journal.pcbi.1005800)
Supplement: S4 Fig — Umbrella histograms along the weight of the open state, here taken from the two-state refinement of LBP against the open/closed 50:50 SAXS curve. (PDF) [file pcbi.1005800.s004.pdf]

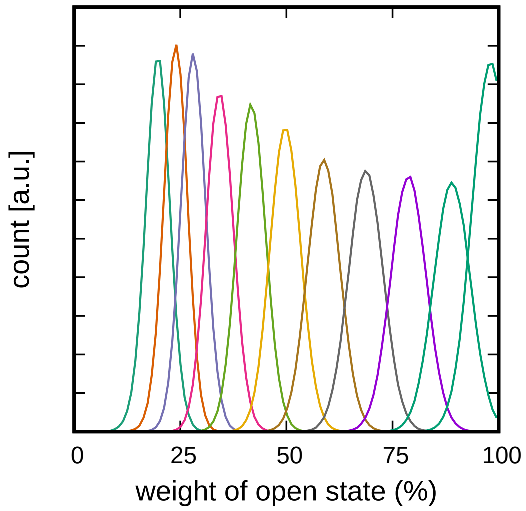

Figure S4. Example of umbrella histograms. Umbrella histograms along the weight of the open state, here taken from the two-state refinement of LBP against the open/closed 50:50 SAXS curve.
