# Supplementary material for: Bayesian refinement of protein structures and ensembles against SAXS data using molecular dynamics
Source: PLoS Comput Biol. 2017 Oct 18;13(10):e1005800. doi: 10.1371/journal.pcbi.1005800 (PMC5662244; doi:10.1371/journal.pcbi.1005800)
Supplement: S5 Fig — Root mean-square deviation (RMSDs) between the Cα atoms of (i) the refined open structure of LBP and (ii) the open LBP structure (similar to Fig 2B). For the RMSD calculations, the open structure was taken as the average structure of the ensembles used to compute the SAXS curve of the open state. Trajectories were taken from umbrella windows of wopen close to the peak of the posterior p(wopen|D, K). The color indicates the open:closed weights used to compute the target curve (see legend, five target curves in Fig 2C). The RMSD curves demonstrate that, starting from the closed state, LBP rapidly opens and approaches the open structure. Some RMSD fluctuations after longer time (green curves) reflect a smaller twist motion between the N- and C-terminal domains within the open state. Such fluctuations along the twist are expected since, as shown previously [18], the SAXS data restrains the degree of openness but not the twist. (PDF) [file pcbi.1005800.s005.pdf]

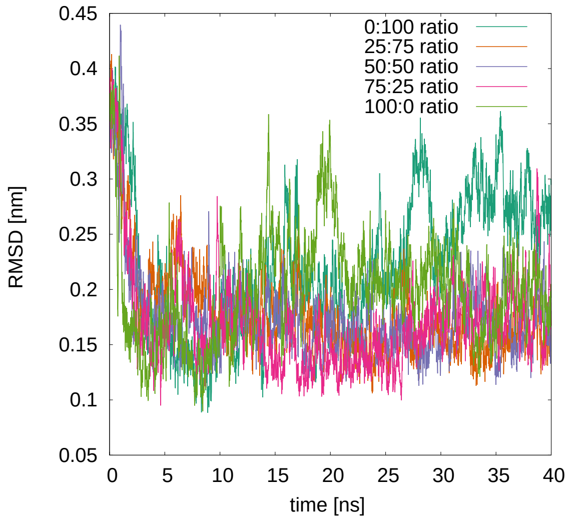

Figure S5. Root mean-square deviation (RMSD) analysis of LBP. Root mean-square deviation (RMSDs) between the C_α atoms of (i) the refined open structure of LBP and (ii) the open LBP structure (similar to Fig. 2B). For the RMSD calculations, the open structure was taken as the average structure of the ensembles used to compute the SAXS curve of the open state. Trajectories were taken from umbrella windows of w_{open} close to the peak of the posterior $p(w_{\mathrm{open}}|D, K)$. The color indicates the open:closed weights used to compute the target curve (see legend, five target curves in Fig. 2C). The RMSD curves demonstrate that, starting from the closed state, LBP rapidly opens and approaches the open structure. Some RMSD fluctuations after longer time (green curves) reflect a smaller twist motion between the N- and C-terminal domains within the open state. Such fluctuations along the twist are expected since, as shown previously (Chen and Hub, Biophys J. 2015), the SAXS data restrains the degree of openness but not the twist.
