# Supplementary material for: Bayesian refinement of protein structures and ensembles against SAXS data using molecular dynamics
Source: PLoS Comput Biol. 2017 Oct 18;13(10):e1005800. doi: 10.1371/journal.pcbi.1005800 (PMC5662244; doi:10.1371/journal.pcbi.1005800)
Supplement: S6 Fig — (A/B) Marginalized posteriors for LBP refined against the SAXS curve with 50:50 open/closed weight (Fig 2D, middle panel), computed from time bins as indicated in the legend. (A) Posterior of the interdomain distance dNC and (B) of the weight of the open state wopen. (C) Marginalized posteriors of wopen for different liganded states of Hsp90 as indicated in the legend. The thin lines indicate posteriors computed from an increasing number of histograms: 10 equally spaced histograms, the same 10 plus additional three histograms near the posterior maximum, 10 plus 6 additional histograms, and 10 plus 9 additional histograms near the posterior maximum. The similarity between the posteriors suggest that the posteriors are reasonably converged. (PDF) [file pcbi.1005800.s006.pdf]

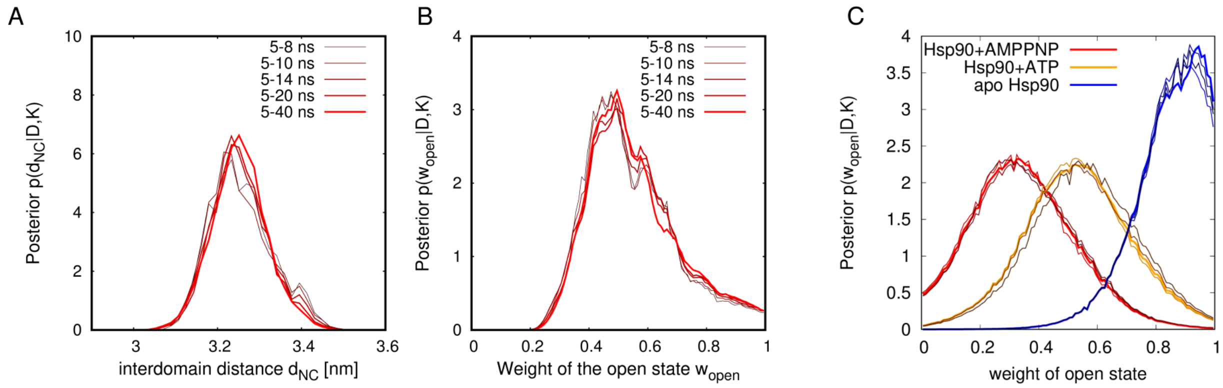

Figure S6. Analysis of the convergence of posterior distributions with increasing invested simulation time. (A/B) Marginalized posteriors for LBP refined against the SAXS curve with 50:50 open/closed weight (Fig. 2D, middle panel), computed from time bins as indicated in the legend. (A) Posterior of the interdomain distance d_{NC} and (B) of the weight of the open state w_{open}. (C) Marginalized posteriors of w_{open} for different liganded states of Hsp90 as indicated in the legend. The thin lines indicate posteriors computed from an increasing number of histograms: 10 equally spaced histograms, the same 10 plus additional three histograms near the posterior maximum, 10 plus 6 additional histograms, and 10 plus 9 additional histograms near the posterior maximum. The similarity between the posteriors suggest that the posteriors are reasonably converged.
