# Supplementary material for: Bayesian refinement of protein structures and ensembles against SAXS data using molecular dynamics
Source: PLoS Comput Biol. 2017 Oct 18;13(10):e1005800. doi: 10.1371/journal.pcbi.1005800 (PMC5662244; doi:10.1371/journal.pcbi.1005800)
Supplement: S1 Table — All numbers in %. The respective posteriors are shown in Fig 3A. (PDF) [file pcbi.1005800.s007.pdf]

True w_{open}	maximum	65% interval		95% interval	
0	0	0	6	0	35
25	24	17	42	11	79
50	50	39	68	30	92
75	70	56	86	44	97
100	100	83	100	67	100

Table S1: Maxima and confidence intervals of w_{open}, taken from $p(w_{open}|D, K)$ of the two-state ensemble refinement of LBP. All numbers in %. The respective posteriors are shown in Fig. 3A of the main text.
