# Supplementary material for: Bayesian refinement of protein structures and ensembles against SAXS data using molecular dynamics
Source: PLoS Comput Biol. 2017 Oct 18;13(10):e1005800. doi: 10.1371/journal.pcbi.1005800 (PMC5662244; doi:10.1371/journal.pcbi.1005800)
Supplement: S2 Table — During two-state refinement, in simulations with non-zero wopen, the posterior of the interdomain distance p(dNC|D, K) of the open state peaks at the physically correct dNC ≈ 3.25 nm. The respective posteriors are shown in S1A Fig. All distances are in nanometers. (PDF) [file pcbi.1005800.s008.pdf]

Table S2: Maxima and confidence intervals of d_{NC}, taken from $p(d_{\mathrm{NC}}|D, K)$ of the two-state ensemble refinement of LBP. All interdomain distances d_{NC} in nanometer. During two-state refinement, in simulations with non-zero w_{open}, the posterior of the interdomain distance $p(d_{\mathrm{NC}}|D, K)$ of the open state peaks at the physically correct $d_{\mathrm{NC}} \approx 3.25\,\mathrm{nm}$. The respective posteriors are shown in Fig. S1A.

True w_{open} (%)	True mean d_{NC}	maximum	65% interval		95% interval	
0	3.25	3.38	3.21	3.44	3.01	3.48
25	3.25	3.23	3.15	3.33	3.06	3.42
50	3.25	3.25	3.17	3.33	3.12	3.40
75	3.25	3.25	3.19	3.33	3.15	3.40
100	3.25	3.27	3.21	3.35	3.15	3.42
