# Supplementary material for: Bayesian refinement of protein structures and ensembles against SAXS data using molecular dynamics
Source: PLoS Comput Biol. 2017 Oct 18;13(10):e1005800. doi: 10.1371/journal.pcbi.1005800 (PMC5662244; doi:10.1371/journal.pcbi.1005800)
Supplement: S3 Table — Refining a single state against SAXS curves that, in truth, represent a heterogenous ensemble of open/closed states, yields posterior distributions that peak at the “mean” interdomain distance 〈dNC〉=wopendNCopen+(1-wopen)dNCclosed, where dNCopen and dNCclosed denote the mean interdomain distances of the open and closed states, in free simulations, respectively. The respective posteriors are shown in Fig 1B. All distances are in nanometers. (PDF) [file pcbi.1005800.s009.pdf]

Table S3: Maxima and confidence intervals of d_{NC} taken from $p(d_{\mathrm{NC}}|D, K)$ of the single-state refinement of LBP. All interdomain distances d_{NC} in nanometer. Refining a single state against SAXS curves that, in truth, represent a heterogenous ensemble of open/closed states, yields posterior distributions that peak at the "mean" interdomain distance $\langle d_{\mathrm{NC}} \rangle = w_{\mathrm{open}} d_{\mathrm{NC}}^{\mathrm{open}} + (1 - w_{\mathrm{open}}) d_{\mathrm{NC}}^{\mathrm{closed}}$, where $d_{\mathrm{NC}}^{\mathrm{open}}$ and $d_{\mathrm{NC}}^{\mathrm{closed}}$ denote the mean interdomain distances of the open and closed states, in free simulations, respectively. The respective posteriors are shown in Fig. S1B.

True w_{open} (%)	mean $\langle d_{\mathrm{NC}} \rangle$	maximum	65% interval		95% interval	
0	3	3.01	2.96	3.06	2.93	3.12
25	3.0625	3.12	3.07	3.16	3.03	3.19
50	3.125	3.14	3.10	3.20	3.06	3.24
75	3.1875	3.20	3.16	3.24	3.11	3.28
100	3.25	3.23	3.20	3.28	3.16	3.34
