# Supplementary material for: Bayesian refinement of protein structures and ensembles against SAXS data using molecular dynamics
Source: PLoS Comput Biol. 2017 Oct 18;13(10):e1005800. doi: 10.1371/journal.pcbi.1005800 (PMC5662244; doi:10.1371/journal.pcbi.1005800)
Supplement: S4 Table — All numbers in %. The respective posteriors are shown in Fig 4D of the main text. (PDF) [file pcbi.1005800.s010.pdf]

Table S4: Maxima and confidence intervals of w_{open} taken from $p(w_{\text{open}}|D, K)$ of the two-state ensemble refinement of Hsp90. All numbers in %. The respective posteriors are shown in Fig. 4D of the main text.

System	maximum	65% interval		95% interval	
Hsp90+AMPPNP	32	17	51	4	73
Hsp90+ATP	52	35	71	16	90
apo Hsp90	94	78	100	59	100
