# Supplementary material for: Bayesian refinement of protein structures and ensembles against SAXS data using molecular dynamics
Source: PLoS Comput Biol. 2017 Oct 18;13(10):e1005800. doi: 10.1371/journal.pcbi.1005800 (PMC5662244; doi:10.1371/journal.pcbi.1005800)
Supplement: S1 Archive — (BZ2) [file pcbi.1005800.s011.tar.bz2 › gromacs_4.62_bayesian_saxs_refi/admin/installguide/installguide.xhtml]

xml version="1.0" encoding="utf-8"?


GROMACS installation guide


- GROMACS installation guide
  - 1 Building GROMACS
  - 2 Prerequisites
    - 2.1 Platform
    - 2.2 Compiler
      - 2.2.1 Running in parallel
    - 2.3 CMake
    - 2.4 Fast Fourier Transform library
      - 2.4.1 FFTW
      - 2.4.2 MKL
    - 2.5 Optional build components
  - 3 Doing a build of GROMACS
    - 3.1 Configuring with CMake
    - 3.2 Using CMake command-line options
    - 3.3 CMake advanced options
    - 3.4 Helping CMake find the right libraries/headers/programs
    - 3.5 CMake advice during the GROMACS 4.6 beta phase
    - 3.6 Native GPU acceleration
    - 3.7 Static linking
    - 3.8 Suffixes for binaries and libraries
    - 3.9 Building GROMACS
    - 3.10 Installing GROMACS
    - 3.11 Getting access to GROMACS after installation
    - 3.12 Testing GROMACS for correctness
    - 3.13 Testing GROMACS for performance
    - 3.14 Having difficulty?
  - 4 Special instructions for some platforms
    - 4.1 Building on Windows
    - 4.2 Building on Cray
    - 4.3 Building on BlueGene
      - 4.3.1 BlueGene/P
      - 4.3.2 BlueGene/Q
  - 5 Tested platforms
  - 6 Other issues

# GROMACS installation guide

## 1. Building GROMACS

These instructions pertain to building GROMACS 4.6 beta releases
and newer. For installations instructions for old GROMACS versions,
see here
http://www.gromacs.org/Documentation/Installation\_Instructions\_4.5.

## 2. Prerequisites

### 2.1. Platform

GROMACS can be compiled for any distribution of Linux, Mac OS X,
Windows (native, Cygwin or MinGW), BlueGene, Cray and probably others.
Technically, it can be compiled on any platform with an ANSI C
compiler and supporting libraries, such as the GNU C library. It can
even compile on an iPhone! Later, there will be a detailed list of
hardware, platform and compilers upon which we do build and regression
testing.

### 2.2. Compiler

GROMACS requires an ANSI C compiler that complies with the C89
standard. For best performance, the GROMACS team strongly
recommends you get the most recent version of your preferred compiler
for your platform (e.g. GCC 4.7 or Intel 12.0 or newer on x86
hardware). There is a large amount of GROMACS code introduced in
version 4.6 that depends on effective compiler optimization to get
high performance - the old assembly-language routines have all
gone. For other platforms, use the vendor's compiler, and check for
specialized information below.

#### 2.2.1. Running in parallel

GROMACS can run in parallel on multiple cores of a single
workstation using its built-in ThreadMPI. No user action is required
in order to enable this.

If you wish to use the excellent new native GPU support in GROMACS,
NVIDIA's CUDA
http://www.nvidia.com/object/cuda\_home\_new.html version
3.2 software development kit is required, and the latest
version is encouraged. NVIDIA GPUs with at least NVIDIA compute
capability 2.0 are required, e.g. Fermi or Kepler cards.

The GPU support from GROMACS version 4.5 using OpenMM
https://simtk.org/home/openmm is still available, also requires
CUDA, and remains the only hardware-based acceleration available
for implicit solvent simulations in GROMACS. This parallelization
path may not be maintained in the future.

If you wish to run in parallel on multiple machines across a network,
you will need to have

- an MPI library installed that supports the MPI 1.3
  standard, and
- wrapper compilers that will compile code using that library.

The GROMACS team recommends OpenMPI
http://www.open-mpi.org/ version 1.4.1 (or higher), MPICH
http://www.mpich.org/ version 1.4.1 (or higher), or your
hardware vendor's MPI installation. The most recent version of
either of this is likely to be the best. LAM/MPI
http://www.lam-mpi.org/ may work, but since it has been
deprecated for years, it is not supported.

In some cases, OpenMP parallelism is an advantage for GROMACS,
but support for this is generally built into your compiler and you
need to make no advance preparation for this. The performance gain you
might achieve can vary with the compiler.

It is important to examine how you will use GROMACS and upon what
hardware and with what compilers in deciding which parallelization
paths to make available. Testing the performance of different options
is unfortunately mandatory. The days of being able to just build and
run '`mdrun`' and get decent performance by default on your
hardware are long gone. GROMACS will always run correctly, and does
a decent job of trying to maximize your performance, but if you want
to approach close to the optimum, you will need to do some work for
it!

### 2.3. CMake

From version 4.6, GROMACS has moved to use the build system
CMake. The previous build system that used configure from
the GNU autotools package has been removed permanently. CMake
permits the GROMACS team to support a very wide range of hardware,
compilers and build configurations while continuing to provide the
portability, robustness and performance for which GROMACS is known.

GROMACS requires CMake version 2.8.0 or higher. Lower
versions will not work. You can check whether CMake is installed,
and what version it is, with cmake --version. If you need to
install CMake, then first check whether your platform's package
management system provides a suitable version, or visit
http://www.cmake.org/cmake/help/install.html for pre-compiled
binaries, source code and installation instructions. The GROMACS
team recommends you install the most recent version of CMake you
can.

### 2.4. Fast Fourier Transform library

Many simulations in GROMACS make extensive use of Fourier transforms,
and a software library to perform these is always required. We
recommend FFTW http://www.fftw.org/ (version 3 or higher
only) or Intel's MKL
http://software.intel.com/en-us/intel-mkl.

#### 2.4.1. FFTW

FFTW is likely to be available for your platform via its package
management system, but there can be compatibility and significant
performance issues associated with these packages. In particular,
GROMACS simulations are normally run in single floating-point
precision whereas the default FFTW package is normally in double
precision, and good compiler options to use for FFTW when linked to
GROMACS may not have been used. Accordingly, the GROMACS team
recommends either

- that you permit the GROMACS installation to download and
  build FFTW 3.3.2 from source automatically
  for you, or
- that you build FFTW from the source code.

If you build FFTW from source yourself, get the most recent version
and follow its installation guide
(e.g. http://www.fftw.org/fftw3\_doc/Installation-and-Customization.html). Choose
the precision (i.e. single or float vs double) to match what you will
later require for GROMACS. There is no need to compile with
threading or MPI support, but it does no harm. On x86 hardware,
compile *only* with --enable-sse2 (regardless of
precision) even if your processors can take advantage of AVX
extensions to SSE. The way GROMACS uses Fourier transforms
cannot take advantage of this feature in FFTW because of memory
system performance limitations, it can degrade performance by around
20%, and there is no way for GROMACS to require the use of the
SSE2 at run time if AVX support has been compiled into FFTW.

#### 2.4.2. MKL

Using MKL requires a set of linker flags that GROMACS is not
able to detect for you, so setting up optimal linking is tricky at the
moment. Need better documentation later.

### 2.5. Optional build components

- A hardware-optimized BLAS or LAPACK library is useful for
  some of the GROMACS utilities, but is not needed for running
  simulations.
- The built-in GROMACS trajectory viewer ngmx requires
  X11 and Motif/Lesstif libraries and header files. Generally, the
  GROMACS team recommends you use third-party software for
  visualization, such as VMD
  http://www.ks.uiuc.edu/Research/vmd/ or PyMOL
  http://www.pymol.org/.

## 3. Doing a build of GROMACS

This section will cover a general build of GROMACS with CMake,
but it is not an exhaustive discussion of how to use CMake. There
are many resources available on the web, which we suggest you search
for when you encounter problems not covered here. The material below
applies specifically to builds on Unix-like systems, including Linux,
Mac OS X, MinGW and Cygwin. For other platforms, see the specialist
instructions below.

### 3.1. Configuring with CMake

CMake will run many tests on your system and do its best to work
out how to build GROMACS for you. If you are building GROMACS on
hardware that is identical to that where you will run mdrun,
then you can be sure that the defaults will be pretty good. Howver, if
you want to control aspects of the build, there's plenty of things you
can set, too.

The best way to use CMake to configure GROMACS is to do an
“out-of-source” build, by making another directory from which you
will run CMake. This can be a subdirectory or not, it doesn't
matter. It also means you can never corrupt your source code by trying
to build it! So, the only required argument on the CMake command
line is the name of the directory containing the
CMakeLists.txt file of the code you want to build. For
example, download the source tarball and use

```
$ tar xfz gromacs-4.6-beta1-src.tgz
$ cd gromacs-4.6-beta1
$ mkdir build-cmake
$ cd build-cmake
$ cmake ..
```

You will see cmake report the results of a large number of
tests on your system made by CMake and by GROMACS. These are
written to the CMake cache, kept in CMakeCache.txt. You
can edit this file by hand, but this is not recommended because it is
easy to reach an inconsistent state. You should not attempt to move or
copy this file to do another build, because the paths are hard-coded
within it. If you mess things up, just delete this file and start
again with '`cmake`'.

If there's a serious problem detected at this stage, then you will see
a fatal error and some suggestions for how to overcome it. If you're
not sure how to deal with that, please start by searching on the web
(most computer problems already have known solutions!) and then
consult the gmx-users mailing list. There are also
informational warnings that you might like to take on board or
not. Piping the output of cmake through less or
tee can be useful, too.

CMake works in an iterative fashion, re-running each time a setting
is changed to try to make sure other things are consistent. Once
things seem consistent, the iterations stop. Once cmake
returns, you can see all the settings that were chosen and information
about them by using

```
$ ccmake ..
```

Check out http://www.cmake.org/cmake/help/runningcmake.html for
general advice on what you are seeing and how to navigate and change
things. The settings you might normally want to change are already
presented. If you make any changes, then ccmake will notice
that and require that you re-configure (using '`c`'), so that it
gets a chance to make changes that depend on yours and perform more
checking. This might require several configuration stages when you are
using ccmake - when you are using cmake the
iteration is done behind the scenes.

A key thing to consider here is the setting of
GMX\_INSTALL\_PREFIX. You will need to be able to write to this
directory in order to install GROMACS later, and if you change your
mind later, changing it in the cache triggers a full re-build,
unfortunately. So if you do not have super-user privileges on your
machine, then you will need to choose a sensible location within your
home directory for your GROMACS installation.

When cmake or ccmake have completed iterating, the
cache is stable and a build tree can be generated, with '`g`' in
ccmake or automatically with cmake.

### 3.2. Using CMake command-line options

Once you become comfortable with setting and changing options, you
may know in advance how you will configure GROMACS. If so, you can
speed things up by invoking cmake with a command like:

```
$ cmake .. -DGMX_GPU=ON -DGMX_MPI=ON -DGMX_INSTALL_PREFIX=/home/marydoe/programs
```

to build with GPUs, MPI and install in a custom location. You can even
save that in a shell script to make it even easier next time. You can
also do this kind of thing with ccmake, but you should avoid
this, because the options set with '`-D`' will not be able to be
changed interactively in that run of ccmake.

### 3.3. CMake advanced options

The options that can be seen with ccmake are ones that we
think a reasonable number of users might want to consider
changing. There are a lot more options available, which you can see by
toggling the advanced mode in ccmake on and off with
'`t`'. Even there, most of the variables that you might want to
change have a '`CMAKE_`' or '`GMX_`' prefix.

### 3.4. Helping CMake find the right libraries/headers/programs

If libraries are installed in non-default locations their location can
be specified using the following environment variables:

- CMAKE\_INCLUDE\_PATH for header files
- CMAKE\_LIBRARY\_PATH for libraries
- CMAKE\_PREFIX\_PATH for header, libraries and binaries
  (e.g. '`/usr/local`').

The respective '`include`', '`lib`', or '`bin`' is
appended to the path. For each of these variables, a list of paths can
be specified (on Unix seperated with ”:”). Note that these are
enviroment variables (and not CMake command-line arguments) and in
a '`bash`' shell are used like:

```
$ CMAKE_PREFIX_PATH=/opt/fftw:/opt/cuda cmake ..
```

The CC and CXX environment variables are also useful
for indicating to CMake which compilers to use, which can be very
important for maximising GROMACS performance. Similarly,
CFLAGS/CXXFLAGS can be used to pass compiler
options, but note that these will be appended to those set by
GROMACS for your build platform and build type. You can customize
some of this with advanced options such as CMAKE\_C\_FLAGS
and its relatives.

See also: http://cmake.org/Wiki/CMake\_Useful\_Variables#Environment\_Variables

### 3.5. CMake advice during the GROMACS 4.6 beta phase

We'd like users to have the ability to change any setting and still
have the CMake cache stable; ie. not have things you set
mysteriously change, or (worse) the whole thing breaks. We're not
there yet. If you know in advance you will want to use a particular
setting, set that on the initial cmake command line. If you
have to change compilers, do that there, or immediately afterwards in
ccmake. Gross changes like GPU or shared libraries on/off are
more likely to work if you do them on the initial command line,
because that's how we've been doing it while developing and
testing. If you do make a mess of things, there's a great thing about
an out-of-source build - you can just do '`rm -rf *`' and start
again. Easy!

We are interested in learning how you managed to break things. If you
can reproducibly reach a state where CMake can't proceed, or
subsequent compilation/linking/running fails, then we need to know so
we can fix it!

### 3.6. Native GPU acceleration

If you have the CUDA SDK installed, you can use CMake
with:

```
cmake .. -DGMX_GPU=ON -DCUDA_TOOLKIT_ROOT_DIR=/usr/local/cuda
```

(or whichever path has your installation). Note that this will require
a working C++ compiler, and in some cases you might need to handle
this manually, e.g. with the advanced option
CUDA\_HOST\_COMPILER.

More documentation needed here - particular discussion of fiddly
details on Windows, Linux and Mac required. Not all compilers on all
systems can be made to work.

### 3.7. Static linking

Dynamic linking of the GROMACS executables will lead to a smaller
disk footprint when installed, and so is the default. However, on some
hardware or under some circumstances you might need to do static
linking. To link GROMACS binaries statically against the internal
GROMACS libraries, set BUILD\_SHARED\_LIBS=OFF. To link
statically against external libraries as well, the
GMX\_PREFER\_STATIC\_LIBS=ON option can be used. Note, that
in general CMake picks up whatever is available, so this option
only instructs CMake to prefer static libraries when both static
and shared are available. If no static version of an external library
is available, even when the aforementioned option is ON, the shared
library will be used. Also note, that the resulting binaries will
still be dynamically linked against system libraries if that is all
that is available.

### 3.8. Suffixes for binaries and libraries

It is sometimes convenient to have different versions of the same
GROMACS libraries installed. The most common use cases have been
single and double precision, and with and without MPI. By default,
GROMACS will suffix binaries and libraries for such builds with
'`_d`' for double precision and/or '`_mpi`' for MPI (and
nothing otherwise). This can be controlled manually with
GMX\_DEFAULT\_SUFFIX, GMX\_BINARY\_SUFFIX and
GMX\_LIBRARY\_SUFFIX.

### 3.9. Building GROMACS

Once you have a stable cache, you can build GROMACS. If you're not
sure the cache is stable, you can re-run `cmake ..` or
`ccmake ..`' to see. Then you can run make to start the
compilation. Before actual compilation starts, make checks
that the cache is stable, so if it isn't you will see CMake run
again.

So long as any changes you've made to the configuration are sensible,
it is expected that the make procedure will always complete
successfully. The tests GROMACS makes on the settings you choose
are pretty extensive, but there are probably a few cases we haven't
thought of yet. Search the web first for solutions to problems,
but if you need help, ask on gmx-users, being sure to provide
as much information as possible about what you did, the system you are
building on, and what went wrong.

If you have a multi-core or multi-CPU machine with N
processors, then using

```
$ make -j N
```

will generally speed things up by quite a bit.

### 3.10. Installing GROMACS

Finally, make install will install GROMACS in the
directory given in GMX\_INSTALL\_PREFIX. If this is an system
directory, then you will need permission to write there, and you
should use super-user privileges only for make install and
not the whole procedure.

### 3.11. Getting access to GROMACS after installation

GROMACS installs the script GMXRC in the bin
subdirectory of the installation directory
(e.g. /usr/local/gromacs/bin/GMXRC), which you should source
from your shell:

```
$ source your-installation-prefix-here/bin/GMXRC
```

It will detect what kind of shell you are running and
set up your environment for using GROMACS. You may wish to arrange
for your login scripts to do this automatically; please search the web
for instructions on how to do this for your shell.

### 3.12. Testing GROMACS for correctness

TODO install and use regression set

### 3.13. Testing GROMACS for performance

TODO benchmarks

### 3.14. Having difficulty?

You're not alone, this can be a complex task. If you encounter a
problem with installing GROMACS, then there are a number of
locations where you can find assistance. It is recommended that you
follow these steps to find the solution:

1. Read the installation instructions again, taking note that you
   have followed each and every step correctly.
2. Search the GROMACS website and users emailing list for
   information on the error.
3. Search the internet using a search engine such as Google.
4. Post to the GROMACS users emailing list gmx-users
   for assistance. Be sure to give a full description of what you have
   done and why you think it didn't work. Give details about the system
   on which you are installing. Copy and paste your command line and as
   much of the output as you think might be relevant - certainly from
   the first indication of a problem. Describe the machine and
   operating system you are running on. People who might volunteer to
   help you do not have time to ask you interactive detailed follow-up
   questions, so you will get an answer faster if you provide as much
   information as you think could possibly help.

## 4. Special instructions for some platforms

### 4.1. Building on Windows

Building on Cygwin/MinGW/etc. works just like Unix. Please see the
instructions above.

Building on Windows using native compilers is rather similar to
building on Unix, so please start by reading the above. Then, download
and unpack the GROMACS source archive. The UNIX-standard
.tar.gz format can be managed on Windows, but you may prefer
to browse ftp://ftp.gromacs.org/pub/gromacs to obtain a
.zip format file, which doesn't need any external tools to
unzip on recent Windows systems. Make a folder in which to do the
out-of-source build of GROMACS. For example, make it within the
folder unpacked from the source archive, and call it “build-cmake”.

Next, you need to open a command shell. If you do this from within
your IDE (e.g. Microsoft Visual Studio), it will configure the
environment for you. If you use a normal Windows command shell, then
you will need to either set up the environment to find your compilers
and libraries yourself, or run the vcvarsall.bat batch script
provided by MSVC (just like sourcing a bash script under
Unix). Presumably Intel's IDE has a similar functionality.

Within that command shell, change to the folder you created above. Run
`cmake ..`, where the folder you point CMake towards is the
folder created by the GROMACS installer. Resolve issues as
above. You will probably make your life easier and faster by using the
new facility to download and install FFTW automatically. After the
initial run of `cmake`, you may wish to use `cmake`,
`ccmake` or the GUI version of CMake until your configuration
is complete.

To compile GROMACS, you then use `cmake --build .` so the
right tools get used.

### 4.2. Building on Cray

Probably you need to build static libraries only? Volunteer needed.

### 4.3. Building on BlueGene

#### 4.3.1. BlueGene/P

Mark to write later. There is currently no native acceleration on this
platform, but the default plain C kernels will work.

#### 4.3.2. BlueGene/Q

Mark to write later. There is currently no native acceleration on this
platform, but the default plain C kernels will work.

## 5. Tested platforms

While it is our best belief that GROMACS will build and run pretty
much everywhere, it's important that we tell you where we really know
it works because we've tested it. We do test on Linux, Windows, and
Mac with a range of compilers and libraries for a range of our
configuration options. Every commit in our git source code
repository is tested on … We test irregularly on…

Contributions to this section are welcome.

Later we might set up the ability for users to contribute test results
to Jenkins.

## 6. Other issues

The GROMACS utility programs often write data files in formats
suitable for the Grace plotting tool, but it is straightforward to
use these files in other plotting programs, too.
